# Plasma Phosphorylated Tau 217 to Identify Preclinical Alzheimer Disease

**DOI:** 10.1001/jamaneurol.2025.3217

**Published:** 2025-09-15

**Authors:** Gemma Salvadó, Shorena Janelidze, Divya Bali, Anna Orduña Dolado, Joseph Therriault, Wagner S. Brum, Alexa Pichet Binette, Erik Stomrud, Niklas Mattsson-Carlgren, Sebastian Palmqvist, Emma M. Coomans, Charlotte E. Teunissen, Wiesje M. van der Flier, Nesrine Rahmouni, Tammie L. S. Benzinger, Juan Domingo Gispert, Kaj Blennow, Vincent Doré, Azadeh Feizpour, Christopher C. Rowe, Daniel Alcolea, Juan Fortea, Sylvia Villeneuve, Sterling C. Johnson, Pedro Rosa-Neto, Ronald C. Petersen, Clifford R. Jack, Suzanne E. Schindler, Marc Suárez-Calvet, Rik Ossenkoppele, Oskar Hansson

**Affiliations:** 1 Clinical Memory Research Unit, Department of Clinical Sciences Malmö, Lund University, Lund, Sweden; 2 Translational Neuroimaging Laboratory, McConnell Brain Imaging Centre, The McGill University Research Centre for Studies in Aging, Montréal, Quebec, Canada; 3 Montreal Neurological Institute, McGill University, Montreal, Quebec, Canada; 4 Department of Psychiatry and Neurochemistry, Institute of Neuroscience and Physiology, The Sahlgrenska Academy, University of Gothenburg, Mölndal, Sweden; 5 Graduate Program in Biological Sciences: Biochemistry, Universidade Federal do Rio Grande do Sul, Porto Alegre, Brazil; 6 Memory Clinic, Skåne University Hospital, Malmö, Sweden; 7 Wallenberg Center for Molecular Medicine, Lund University, Lund, Sweden; 8 Alzheimer Center Amsterdam, Amsterdam University Medical Center, Vrije Universiteit, Amsterdam, the Netherlands; 9 Amsterdam Neuroscience, Brain Imaging, Amsterdam, the Netherlands; 10 Neurochemistry Laboratory, Amsterdam University Medical Center, Vrije Universiteit Amsterdam, the Netherlands; 11 Amsterdam Neuroscience, Neurodegeneration, Amsterdam, the Netherlands; 12 Epidemiology and Data Science, Amsterdam University Medical Center, Amsterdam, the Netherlands; 13 Washington University School of Medicine in St Louis, St Louis, Missouri; 14 Knight Alzheimer Disease Research Center, St Louis, Missouri; 15 Barcelonaßeta Brain Research Center, Barcelona, Spain; 16 Centro de Investigación Biomédica en Red Bioingeniería, Biomateriales y Nanomedicina, Instituto de Salud Carlos III, Madrid, Spain; 17 Universitat Pompeu Fabra, Barcelona, Spain; 18 Hospital del Mar Medical Research Institute, Barcelona, Spain; 19 Centro Nacional de Investigaciones Cardiovasculares, Madrid, Spain; 20 Clinical Neurochemistry Laboratory, Sahlgrenska University Hospital, Mölndal, Sweden; 21 Department of Psychiatry and Neurochemistry, Institute of Neuroscience and Physiology, The Sahlgrenska Academy at the University of Gothenburg, Gothenburg, Sweden; 22 Paris Brain Institute, ICM, Pitié-Salpêtrière Hospital, Sorbonne University, Paris, France; 23 Neurodegenerative Disorder Research Center, Division of Life Sciences and Medicine, and Department of Neurology, Institute on Aging and Brain Disorders, University of Science and Technology of China and First Affiliated Hospital of the University of Science and Technology of China, Hefei, People’s Republic of China; 24 Department of Molecular Imaging & Therapy, Austin Health, Melbourne, Victoria, Australia; 25 The Florey Institute of Neuroscience and Mental Health, The University of Melbourne, Parkville, Victoria, Australia; 26 Sant Pau Memory Unit, Department of Neurology, Institut d’Investigació Biomèdica Sant Pau, Hospital de La Santa Creu I Sant Pau, Barcelona, Spain; 27 Centro de Investigación Biomédica en Red en Enfermedades Neurodegenerativas, Centro de Investigación Biomédica en Red en Enfermedades Neurodegenerativas, Madrid, Spain; 28 Barcelona Down Medical Center, Fundació Catalana Síndrome de Down, Barcelona, Spain; 29 Centre for Studies on Prevention of Alzheimer’s Disease, Douglas Mental Health Institute, Montreal, Quebec, Canada; 30 Department of Psychiatry, Faculty of Medicine, McGill University, Montreal, Quebec, Canada; 31 Wisconsin Alzheimer’s Disease Research Center, University of Wisconsin-Madison School of Medicine and Public Health, Madison; 32 Wisconsin Alzheimer’s Institute, University of Wisconsin School of Medicine and Public Health, Madison; 33 Centre for Studies on Prevention of Alzheimer’s Disease, Montreal, Quebec, Canada; 34 Department of Neurology, Mayo Clinic, Rochester, Minnesota; 35 Department of Radiology, Mayo Clinic, Rochester, Minnesota; 36 Servei de Neurologia, Hospital del Mar, Barcelona, Spain; 37 Alzheimer Center Amsterdam, Neurology, Amsterdam University Medical Center, Amsterdam, the Netherlands; 38 The Australian e-Health Research Centre, Commonwealth Scientific and Industrial Research Organisation, Melbourne, Victoria, Australia; 39 Department of Molecular Imaging and Therapy, Austin Health, Melbourne, Victoria, Australia; 40 Florey Department of Neuroscience and Mental Health, The University of Melbourne, Melbourne, Victoria, Australia

## Abstract

**Importance:**

Advances in Alzheimer disease (AD) have shifted research focus to earlier disease stages, necessitating more scalable approaches to identify cognitively unimpaired individuals with amyloid β (Aβ) pathology.

**Objective:**

To assess the utility of plasma phosphorylated tau 217 (p-tau217) for classifying Aβ status in cognitively unimpaired individuals, both as a stand-alone test and in a 2-step approach where positive plasma results were confirmed using a second modality (Aβ positron emission tomography [PET] or cerebrospinal fluid [CSF]).

**Design, Setting, and Participants:**

This cross-sectional cohort study used data collected between June 2009 and March 2024. We included 2916 cognitively unimpaired participants from 12 international independent observational cohorts in the US, Europe, Australia, and Canada with available plasma p-tau217 levels and CSF or PET Aβ biomarkers. Performance comparisons between mass spectrometry and immunoassay-based p-tau217 measurements were also performed (n = 964).

**Exposures:**

Plasma p-tau217 levels measured by immunoassay.

**Main Outcome and Measures:**

Aβ status, determined by CSF or Aβ PET biomarkers.

**Results:**

Participants had a mean (SD) age of 66.9 (9.9) years; 971 (33.3%) were Aβ positive by either CSF or PET, 1667 (57.2%) were women, and 1108 (38.1%) carried at least 1 *APOE *ε4 allele. As a stand-alone test, plasma p-tau217 achieved a positive predictive value (PPV) of 79% (95% CI, 74-84) and an overall accuracy of 81% (95% CI, 80-82). In a 2-step workflow, the PPV and accuracy significantly increased to 91% (95% CI, 86-95). While this approach required screening of 677 individuals with plasma p-tau217 to identify 100 Aβ-positive individuals, compared to 536 participants when using PET alone, it reduced the need for PET testing to 124. Immunoassays demonstrated comparable PPVs to mass spectrometry (80% [95% CI, 74-86] vs 85% [95% CI, 81-90]; *P* = .12) but significantly lower overall accuracy (82% [95% CI, 79-84]% vs 88 [95% CI, 86-90]; *P* < .001) and true Aβ-positive detection rate (49% [95% CI, 43-55] vs 69% [95% CI, 64-75]; *P* < .001).

**Conclusions and Relevance:**

The findings highlight the potential of plasma p-tau217 as a stand-alone test—or when used in a sequential 2-step approach alongside PET or CSF testing—as a cost-effective, scalable, and minimally burdensome strategy for identifying preclinical AD. Tailored screening workflows that incorporate p-tau217 can improve efficiency in participant selection for preclinical AD trials and, in the future, help guide access to disease-modifying treatments.

## Key Points

**Question:**

Can plasma phosphorylated tau 217 (p-tau217) reliably identify amyloid β (Aβ) status in cognitively unimpaired individuals for participant selection in preclinical Alzheimer disease (AD) trials or for potential future use in clinical practice?

**Findings:**

In this cohort study of 2196 cognitively unimpaired individuals, plasma p-tau217 demonstrated good accuracy in predicting Aβ positivity. However, achieving a positive predictive value above 90% required confirmatory testing with a cerebrospinal fluid or positron emission tomography test.

**Meaning:**

Plasma p-tau217 is a useful stand-alone marker of Aβ status in cognitively unimpaired individuals when moderate accuracy suffices, but adding another test in a second step is needed to achieve a high accuracy in this population.

## Teaser

This cohort study evaluates the use of plasma phosphorylated tau 217 in identifying preclinical Alzheimer disease in individuals with amyloid β pathology.

## Introduction

Alzheimer disease (AD) is the leading cause of dementia, accounting for 60% to 70% of the estimated 55 million dementia cases worldwide.^1^ There is an urgent need for effective treatments to halt or slow disease progression. Biologically, AD is characterized by the accumulation of amyloid β (Aβ) plaques and tau tangles in the brain. Recent advances in antiamyloid immunotherapies have shown promise in slowing cognitive decline,^2^ and the first disease-modifying treatments have now been approved in several countries. However, these therapies offer only modest clinical benefits in symptomatic AD, highlighting the need for further improvements in AD treatment.^5^

Since Aβ pathology emerges decades before the onset of symptoms,^6^ targeting the disease at earlier stages—when pathology is present, but symptoms and neurodegeneration remain minimal—could enhance the therapeutic benefits of antiamyloid treatment.^7^ This hypothesis is supported by post hoc analyses from recent trials, which indicate more favorable outcomes in participants who were included at earlier clinical and biological stages. Specifically, AD patients at the mild cognitive impairment stage demonstrated better responses than those with dementia.^2^ In theory, therapies may be more effective in cognitively unimpaired older adults with AD pathology. However, identifying cognitively unimpaired individuals with early AD pathology remains challenging. For instance, the Anti-Amyloid Treatment in Asymptomatic AD (A4) study^9^ required more than 4400 people undergoing positron emission tomography (PET) scans and more than 3 years to meet their recruitment goal of 1150 Aβ-positive participants.

Plasma biomarkers offer a potential solution for identifying cognitively unimpaired individuals with early AD pathology, as they are more accessible and cost effective and less burdensome than cerebrospinal fluid (CSF) or PET-based biomarkers.^10^ Among the currently available plasma biomarkers, plasma phosphorylated tau 217 (p-tau217) has consistently shown the highest accuracy in detecting AD pathology.^11^ While its association with AD pathology is well established, its utility in cognitively unimpaired populations is less clear.^17^ This distinction is critical, as the lower prevalence of AD pathology in cognitively unimpaired compared to cognitively impaired populations reduces the positive predictive value (PPV) of a diagnostic test.^23^ Additionally, the lower burden of pathology in cognitively unimpaired individuals further complicates its detection, even with highly accurate tests. Moreover, many studies involving cognitively unimpaired individuals have been relatively small and relied on single assays measured in single batches, limiting the generalizability of findings.^17^

This study aimed to assess the utility of plasma p-tau217 for classifying Aβ status in cognitively unimpaired participants, using data from multiple sites across continents. Our objective was to evaluate the accuracy of plasma p-tau217, both as a stand-alone biomarker and in a 2-step workflow where positive plasma p-tau217 results were confirmed using PET or CSF. An improved workflow could improve efficiency in participant selection for preclinical AD trials, and help guide access to disease-modifying treatments in the future.

## Methods

### Participants

We analyzed data from June 2009 to March 2024 on 2726 cognitively unimpaired participants across 12 independent cohorts in the US, Europe, Australia, and Canada: Amsterdam Dementia Cohort (ADC; n = 46),^25^ Alzheimer’s Disease Neuroimaging Initiative (ADNI; n = 241), Australian Imaging Biomarker and Lifestyle (AIBL; n = 180),^26^ Alzheimer and Families (ALFA; n = 359),^27^ BioFINDER-1 (n = 105),^28^ BioFINDER-2 (n = 595),^28^ Knight Alzheimer Disease Research Center (ADRC; n = 383), Mayo Clinic Study of Aging (MCSA; n = 363),^29^ Pre-Symptomatic Evaluation of Experimental or Novel Treatments for Alzheimer Disease (PREVENT-AD; n = 217),^30^ Sant Pau Initiative on Neurodegeneration (SPIN; n = 171),^31^ Translational Biomarkers in Aging and Dementia (TRIAD; n = 103),^32^ and Wisconsin Registry for Alzheimer Prevention (WRAP; n = 153).^33^ Of these, 1747 participants had both Aβ PET and AD CSF biomarkers available. A detailed description of individual cohorts can be found in eTable 1 in Supplement 1. All study participants provided written informed consent, and local institutional review boards approved the studies. This study followed the Standards for Reporting of Diagnostic Accuracy (STARD) reporting guideline.

### Plasma P-tau 217

Methods for plasma p-tau217 quantification are summarized in eTable 2 in Supplement 1. Two immunoassay platforms were used in the primary analyses: Lilly MSD (ADC, ALFA, BioFINDER-1, BioFINDER-2, Knight ADRC, PREVENT-AD, SPIN, and WRAP) and Janssen R&D Simoa (AIBL, ADNI, MCSA, and TRIAD). Additionally, mass spectrometry–based measures were analyzed in 4 cohorts, measured at Washington University (BioFINDER-2 and Knight ADRC) or C2N Diagnostics (ADNI and WRAP) as previously described.^18^ Plasma p-tau217 levels were log10 transformed and *z* scored within each cohort, based on Aβ-negative participants older than 50 years, excluding extreme outliers (<Q1 − 5IQR or >Q3 + 5IQR), to allow harmonization across cohorts.

### Aβ PET

Aβ PET acquisition and preprocessing methods for each cohort have been described (see eTable 3 in Supplement 1 for main acquisition parameters). Four tracers were used for acquiring Aβ PET: carbon 11–labeled Pittsburgh Compound B (^11^C-PiB; Knight ADRC, MCSA, and WRAP), ^18^F-flutemetamol (ALFA, BioFINDER-1, and BioFINDER-2), ^18^F-florbetapir (ADC, ADNI, and Knight ADRC), and ^18^F-NAV4694 (AIBL, PREVENT-AD, and TRIAD). Aβ PET positivity was determined through quantification when possible or via visual assessment (ADC). Quantitative results were standardized to the Centiloid scale^34^ for comparability. In main analyses Aβ PET was assessed as positive if Centiloids were greater than 25 when quantification was available or by visual assessment. This threshold was based on previous studies supporting its utility to detect early signs of aggregated Aβ pathology.^35^ This threshold is also congruent with visual assessment,^37^ and it also showed good accuracy when compared to positivity by CSF AD in our sample defined by cohort-specific thresholds (n = 1711; accuracy, 87%) (eFigure 1A-B in Supplement 1). Sensitivity analyses used a stricter threshold of Centiloids greater than 37, aligned with the inclusion criteria of the AHEAD 3-45 study,^38^ and a lower threshold greater than 12 based on previous studies suggesting that it is the earliest sign of Aβ deposition.^35^

### CSF Biomarkers

CSF biomarker collection and analysis methods have been described previously. Details for each cohort are in eTable 4 in Supplement 1. We assessed Aβ positivity using either the CSF Aβ42/40 ratio (ALFA, BioFINDER-1, BioFINDER-2, Knight ADRC, SPIN, TRIAD, and WRAP) or the p-tau181/Aβ42 ratio (ADC, ADNI, and PREVENT-AD). Cohort-specific thresholds for positivity were applied, as validated previously in each cohort.

### Statistical Analysis

Plasma p-tau217 differences by Aβ status were assessed with *t* test and Cohen *d*. Receiver operating characteristic curves were used to compare the performance of plasma p-tau217 levels against Aβ positivity. We performed 2 main set of analyses using plasma p-tau217 alone or in combination with CSF or PET.

#### Predictive Accuracy of Plasma P-tau217

We evaluated the ability of plasma p-tau217 to predict Aβ positivity using 3 outcomes: Aβ positivity via either CSF or Aβ PET (a single positive result suffices), Aβ positivity via CSF, and Aβ positivity via PET (to overcome the different set of biomarkers available in the different cohorts). This approach allowed robust evaluation of Aβ status across soluble (CSF) and deposited (PET) biomarkers.

Logistic regression models were used to calculate probabilities of Aβ positivity, with age as a covariate. We used age as an additional variable to help our predictive model because is a very easy assessment to obtain, but we also tested other models in the sensitivity analyses. Thresholds for p-tau217 positivity were determined in a part of the sample (30%) using the cutpointr package in R, optimizing sensitivity while targeting high specificity (95% or 97.5%) to maximize PPVs. Note that PPV = (sensitivity × prevalence) / {[sensitivity × prevalence] + [(1 − specificity) × (1 − prevalence)]}, and thus it depends on prevalence. These thresholds were tested in the rest of the sample (70%), assessing PPVs, negative predictive values (NPVs), accuracy, and the proportion of positive cases detected. Bootstrapping (1000 resamples) was used to assess result variability using the boot package in R.

#### Utility of Adding CSF or PET

We assessed the added value of combining plasma p-tau217 with CSF or PET for predicting Aβ positivity through 2 sets of analyses, using either CSF or PET as the reference standard. In the first analysis, with CSF as the reference, we compared 3 strategies: plasma only, PET only, and a 2-step approach in which only plasma-positive individuals underwent PET. In the second analysis, using PET as the reference, we evaluated plasma only, CSF only and a 2-step approach where plasma-positive individuals received CSF testing. This design allowed us to model 2 widely used clinical reference standards for determining Aβ status.

#### Utility for Screening

Additionally, we conducted recruitment simulations for a hypothetical clinical trial aiming to enroll 100 Aβ-positive cognitively unimpaired participants. We compared 5 strategies: CSF only, PET only, plasma only, and 2-step approaches involving plasma followed by either CSF or PET. For each strategy, we estimated (1) the number of individuals that would need to be screened to recruit 100 true Aβ-positive participants, (2) the number of CSF or PET tests required, and (3) the number of participants ultimately enrolled, including those incorrectly classified as Aβ positive. All analyses were performed separately using CSF and PET as the reference standard.

Main analyses were conducted using optimal 95% specificity thresholds, but we also simulated participants screened, CSF/PET tests conducted, and total enrollees for different specificity thresholds (75%-97.5%). Costs were compared across scenarios using plasma:CSF:PET cost ratios of 1:4:16, 1:5:10, 1:6:25, and 1:8:20, due to the large variability in costs among centers.

#### Mass Spectrometry vs Immunoassay

Additionally, comparative analyses were conducted for participants with both immunoassay- and mass spectrometry–based p-tau217 measures. Thresholds were calculated in the full dataset due to the smaller sample size.

#### Additional Analyses

Sensitivity analyses also explored models with different covariates (none, age only or age and *APOE* ε4 carriership), or different quantitative Aβ PET thresholds. We also tested different specificity thresholds in the 2-step approach. Finally, we also did sensitivity analyses by age ranges.

All statistical analyses were performed with R version 4.3.1 (R Foundation). Statistical comparisons were performed using bootstrapping (n = 1000 resamples with replacement). 2-tailed *P* values less than .05 were considered significant. Only one participant with extreme plasma p-tau217 levels (*z* score, 11.3) was excluded, resulting in the final sample of 2916 participants.

## Results

Plasma p-tau217 measurements and either Aβ PET or CSF AD biomarkers were available from 2916 cognitively unimpaired individuals. Cohort-specific participant characteristics are detailed in eTable 5 in Supplement 1. Among these, 1747 participants had both Aβ PET and CSF AD biomarkers, 998 had only Aβ PET, and 171 had only CSF AD biomarkers. Participants had a mean (SD) age of 66.9 (9.9) years; 1667 (57.2%) were women and 1249 (42.8) were men; 1108 (38.1%) carried at least 1 *APOE* ε4 allele (Table). Aβ status was determined as positive if either CSF (using cohort-specific thresholds) or PET (Centiloids >25 or visual assessment; eFigure 1A-B in Supplement 1)^35^ was positive. If both measures were available, a positive result in either was sufficient for positive classification. The prevalence of Aβ positivity was 33.3% (n = 971) in the whole sample and 32.1% (n = 560) in individuals with both Aβ PET and CSF AD biomarkers.

**Table.** Sample Description

Characteristic	CSF or PET (n = 2916)	CSF and PET (n = 1747)
Age, mean (SD), y	66.9 (9.91)	66.3 (9.11)
Sex, No. (%)		
Female	1667 (57.2)	1005 (57.5)
Male	1249 (42.8)	742 (42.5)
*APOE* ε4 carriers, No. (%)	1108 (38.1)	735 (42.1)
No.^a^	2908	1746
P-tau217, *z* score, mean (SD)	0.457 (1.36)	0.492 (1.39)
Aβ positive, No. (%)^b^	971 (33.3)	560 (32.1)
Aβ PET positive, No. (%)	826 (28.3)	355 (20.3)
No.^a^	2745	1747
Centiloids	13.1 (31.8)	11.3 (30.8)
No.^a^	2336	1711
CSF positive, No. (%)	542 (28.3)	531 (30.4)
No.^a^	1918	1747

Abbreviations: Aβ, amyloid β; CSF, cerebrospinal fluid; NA, not applicable; p-tau217, phosphorylated tau 217; PET, positron emission tomography.

^a^ Number of individuals for whom data were available in each respective category.

^b^ Aβ positivity, defined as Centiloid >25 or visual assessment, was determined by either Aβ PET or CSF biomarker positivity.

### Detection of Aβ Status Using Plasma P-tau217

As expected, plasma p-tau217 levels were higher in participants assessed as Aβ positive (eFigure 2A in Supplement 1). Plasma p-tau217, together with age, predicted Aβ positivity in cognitively unimpaired individuals with an area under the curve of 83% (95% CI, 81-85) (eFigure 2B in Supplement 1).

**Figure 1.**
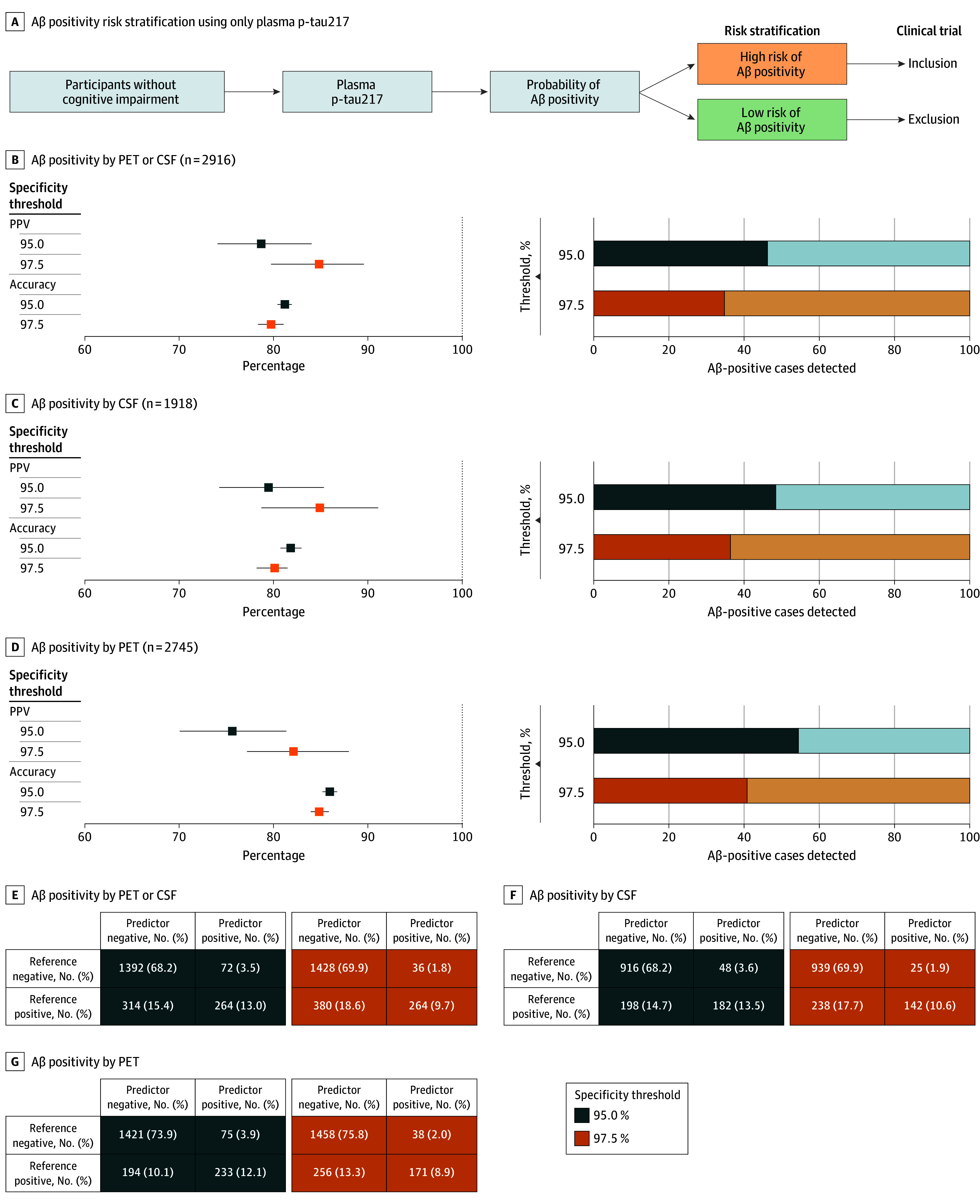
Plasma Phosphorylated Tau 217 (P-tau217) as a Stand-Alone Confirmatory Marker of Amyloid β (Aβ) Positivity A, A schematic overview of the plasma-only approach is depicted. B-D, Clinical accuracy of plasma p-tau217 is shown for 2 specificity thresholds (95% and 97.5%) across different reference standards: Aβ positivity assessed by either Aβ positron emission tomography (PET) or cerebrospinal fluid (CSF) (B), CSF only (C), and Aβ PET only (D). Dashed lines represent the maximum values achievable by a perfect biomarker. The percentage of positive individuals selected by plasma is shaded in the right column. In the bar plots, individuals who would not be selected out of those who were Aβ positive (ie, false-negative rate) are shown in a lighter color. E-G, Cross-tables display predicted vs reference status for each outcome stratified by specificity thresholds. PPV indicates positive predictive value.

When determining an optimal cutoff yielding 95% specificity in the training set, the accuracy of the model was 81% (95% CI, 80-82) with PET or CSF as the reference standard (Figure 1A and B; eTable 6 in Supplement 1) and PPV was 79% (95% CI, 74-84). However, plasma p-tau217 only identified 46% of all Aβ-positive cases as defined by the reference standard. Similar results were observed when CSF alone was used as the reference standard (PPV, 79%; 95% CI, 74-85; accuracy 82%; 95% CI, 81-83; n = 1918) (Figure 1C) and when PET alone was the reference standard (PPV, 76%; 95% CI, 70-81; accuracy 86; 95% CI, 85-87; n = 2745) (Figure 1D).

A more stringent cutoff, yielding 97.5% specificity, achieved higher PPV (85%; 95% CI, 80-90), lower sensitivity (35%; 95% CI, 27-43) but similar accuracy (80%; 95% CI, 78-81) than the 95% specificity threshold. Other specificity thresholds are presented in eFigure 1C in Supplement 1 showing that only very high specificity thresholds (99.5%) could render PPVs higher than 90%.

### Enhanced Detection of Cognitively Unimpaired Aβ-Positive Individuals Using a 2-Step Approach

**Figure 2.**
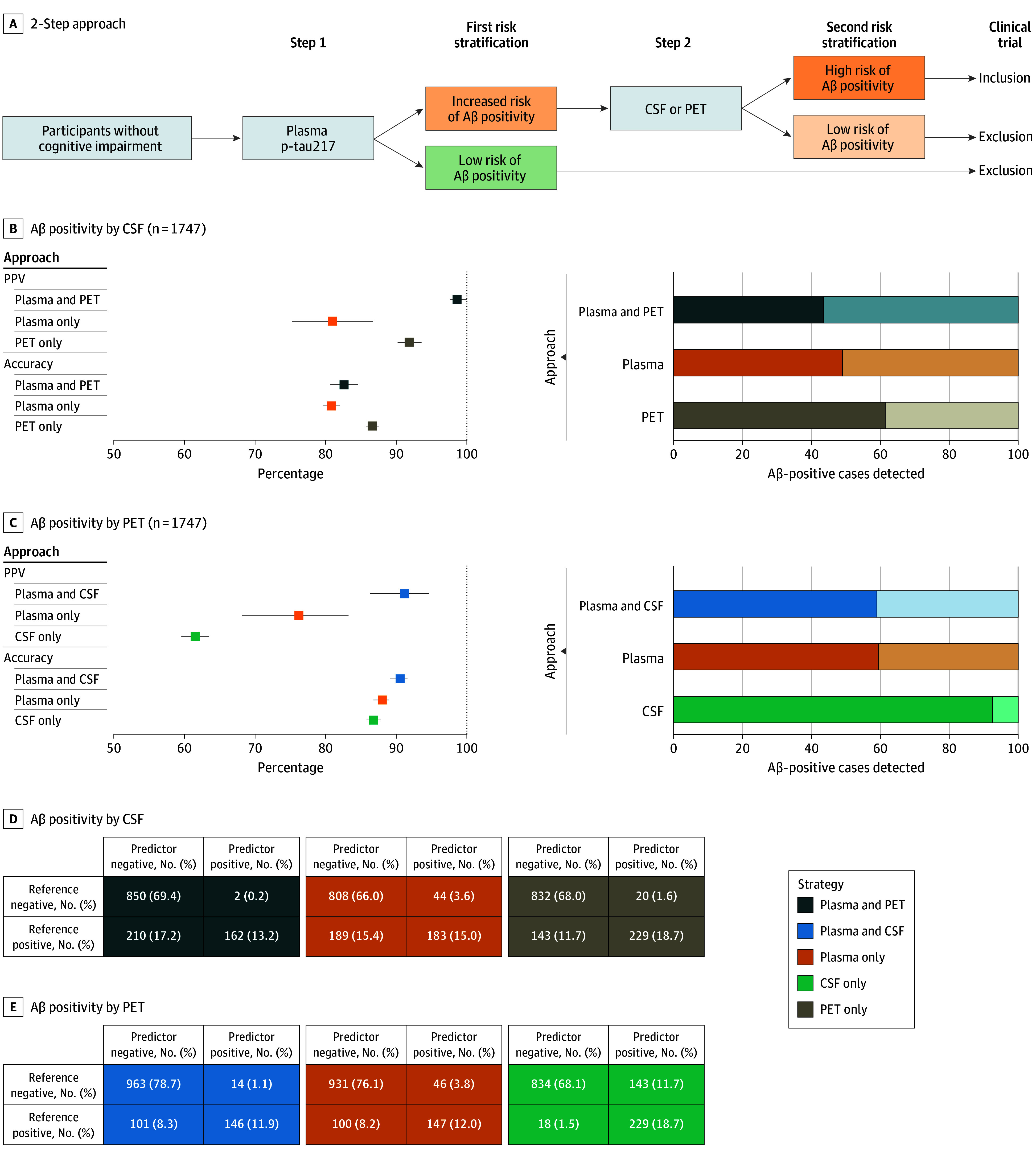
Comparison of Clinical Accuracy Between the 2-Step Approach, Plasma Only, and Cerebrospinal Fluid (CSF) or Positron Emission Tomography (PET) Strategies A, Schematic representation of the 2-step approach. B-C, Statistical results are shown for using CSF (B) or amyloid β (Aβ) PET (C) as the reference standard. Dashed lines indicate the maximum achievable values for a perfect biomarker. The percentage of positive individuals selected by each technique is shaded in the right column. In the bar plots, individuals who would not be selected out of those who were Aβ positive (ie, false-negative rate) are shown in a lighter color. Plasma positivity was determined using a threshold based on 95% specificity across all approaches. D-E, Cross-tables illustrate predicted vs reference status for each outcome, comparing the 3 approaches. P-tau217 indicates phosphorylated tau 217; PPV, positive predictive value.

Next, we aimed to investigate whether the clinical accuracy could be improved by confirming the plasma p-tau217 results with a second biomarker modality (ie, PET or CSF) in participants with a positive plasma p-tau217 result (Figure 2A). This 2-step approach was compared to scenarios using either PET or CSF tests alone or plasma p-tau217 alone.

When Aβ PET was used to confirm the plasma p-tau217 test and CSF was the reference standard (Figure 2B), the PPV was 81% (95% CI, 75-87) for plasma p-tau217 alone, 92% (95% CI, 90-94) for PET alone, and 99% (95% CI, 98-100) for the 2-step approach (positive plasma p-tau217 followed by PET). In the 2-step approach, 73% of individuals with a positive p-tau217 test were also PET positive. Of all CSF-positive participants (reference), only 49% (95% CI, 41-57) were detected using plasma p-tau217 alone, 61% (95% CI, 59-64) with PET alone, and 44% (95% CI, 37-50) with the 2-step approach (eTable 7 in Supplement 1).

We also tested the reversed approach, in which CSF was used to confirm the plasma p-tau217 test and Aβ PET was the reference standard (Figure 2C). Following this approach, PPVs were 76% (95% CI, 68-83) for plasma alone, 62% (95% CI, 60-63) for CSF alone, and 91% (95% CI, 86-95) for the 2-step approach (positive plasma p-tau217 followed by CSF). In this scenario, 82% of plasma-positive cases were CSF positive. Plasma p-tau217 alone detected 60% (95% CI, 50-67) of all PET-positive participants (reference), CSF alone detected 93% (95% CI, 91-94), and the 2-step approach detected 59% (95% CI, 50-66) (eTable 7 in Supplement 1).

The primary advantage of the 2-step approach was a significant reduction in false positives, from 44 (19.4% of the positives) with plasma alone to 2 (0.1% of the positives) with the 2-step model using CSF positivity as reference. From 46 (23.8% of the positives) to 14 (8.8% of the positives) using PET positivity as reference (Figure 2D and E). Comparison of individuals’ characteristics selected by each approach is presented in eFigure 3 in Supplement 1.

### Implications for Preclinical AD Trials

**Figure 3.**
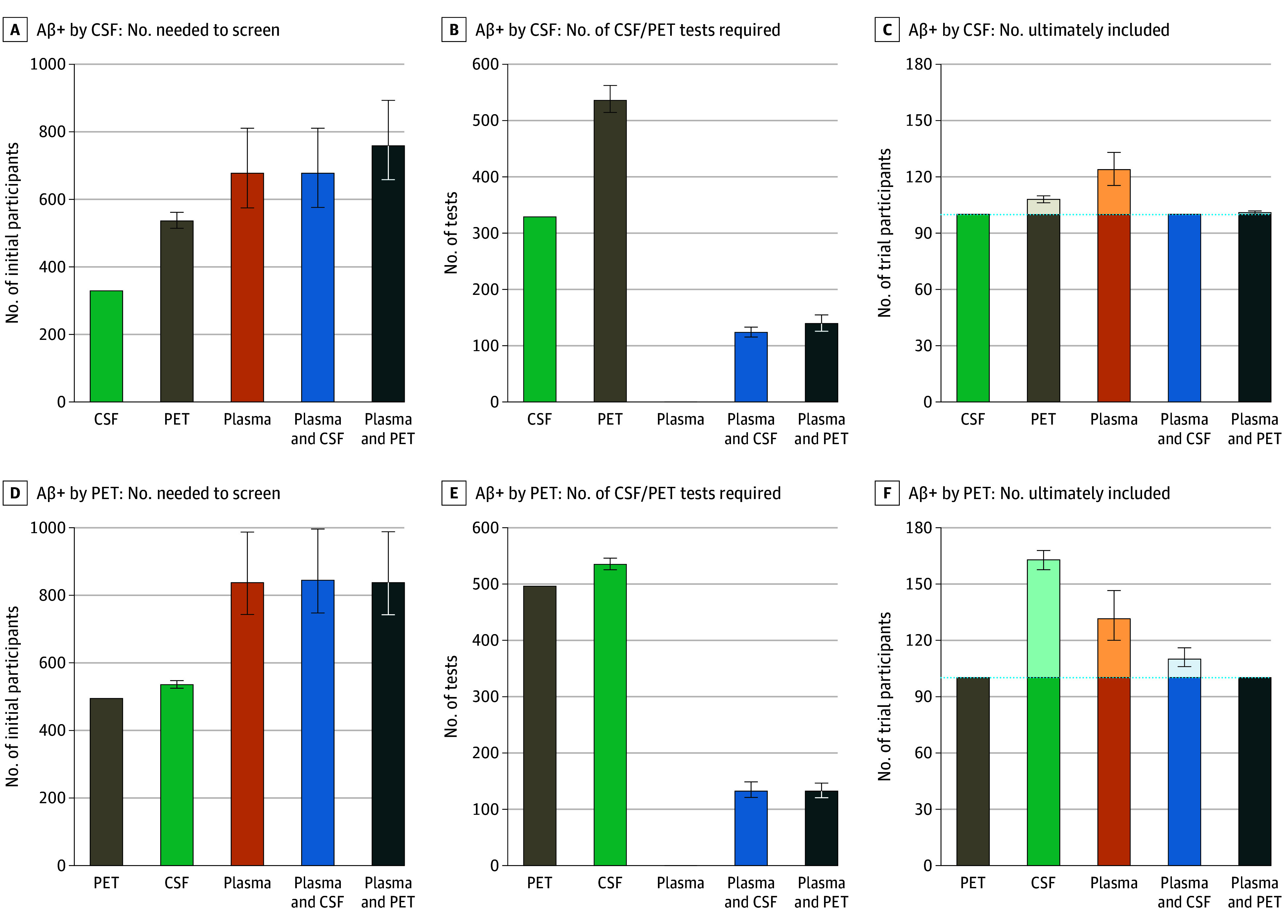
Implications of Different Recruitment Strategies for a Hypothetical Clinical Trial Enrolling 100 Amyloid β (Aβ)-Positive Participants The figure compares the impact of 5 recruitment strategies: positron emission tomography (PET) only, cerebrospinal fluid (CSF) only, plasma only, a 2-step approach using plasma followed by CSF, and a 2-step approach using plasma followed by PET. Results are presented separately using CSF (A-C) or PET (D-F) as the reference standard for Aβ positivity. The first column (A and D) shows the number of individuals that would need to be initially screened under each approach for achieving 100 Aβ-positive individuals. The second column (B and E) shows the number of CSF or PET confirmatory tests required for each approach. The third column (C and F) shows the number of individuals ultimately included in the trial. Individuals who would be included but were Aβ negative (ie, false positives) are shown in a lighter color. Plasma positivity was determined using a threshold set at 95% specificity across all approaches. P-tau217 indicates phosphorylated tau 217.

We next evaluated how the different strategies would impact recruitment for a hypothetical clinical trial aiming to enroll 100 Aβ-positive cognitively unimpaired participants. First, we estimated the number of individuals that would need to be screened under each approach. Using CSF as the reference standard for Aβ status (30% positive), we found that 329 participants would need to be screened when CSF was used for screening, 536 with amyloid PET alone, 677 with plasma p-tau217 alone or plasma followed by CSF, and 760 with plasma followed by PET (Figure 3A).

We then assessed how many CSF or PET tests would be required under each strategy, focusing on the potential benefit of using plasma p-tau217 as an initial screening step. Plasma-based approaches substantially reduced the number of confirmatory tests needed: plasma followed by CSF required lumbar punctures in only 124 individuals, while plasma followed by PET required just 139 PET scans. These figures were significantly lower than the 329 CSF tests or 536 PET scans needed when using CSF or PET alone, respectively (Figure 3B).

Finally, we evaluated how many participants would ultimately be enrolled in the trial. Since neither plasma nor PET perfectly identified CSF-positive individuals, some false positives were included in plasma- or PET-based strategies. The plasma-only approach led to 24 additional participants being enrolled, PET alone added 8, and plasma followed by PET added just 1 extra participant, beyond the target of 100 Aβ-positive cognitively unimpaired individuals (Figure 3C). Similar findings were observed when PET was used as the reference standard instead of CSF (Figures 3D-F).

We also explored how the different approaches would translate into costs of recruitment using different plasma:CSF:PET costs ratios (1:4:16, 1:5:10, 1:6:25, and 1:8:20) due to the large cost differences between centers (eFigure 4 in Supplement 1). In summary, all approaches were more economic relative to Aβ PET alone for recruitment (saved when using CSF: 46%-74%; plasma: 80%-92%; 2-step with CSF: 66%-85%). The plasma-only approaches were the cheapest methods for recruitment in all cases, closely followed by the 2-step approach using plasma followed by CSF.

### Comparison of Mass Spectrometry– and Immunoassay-Based Plasma Measurements

**Figure 4.**
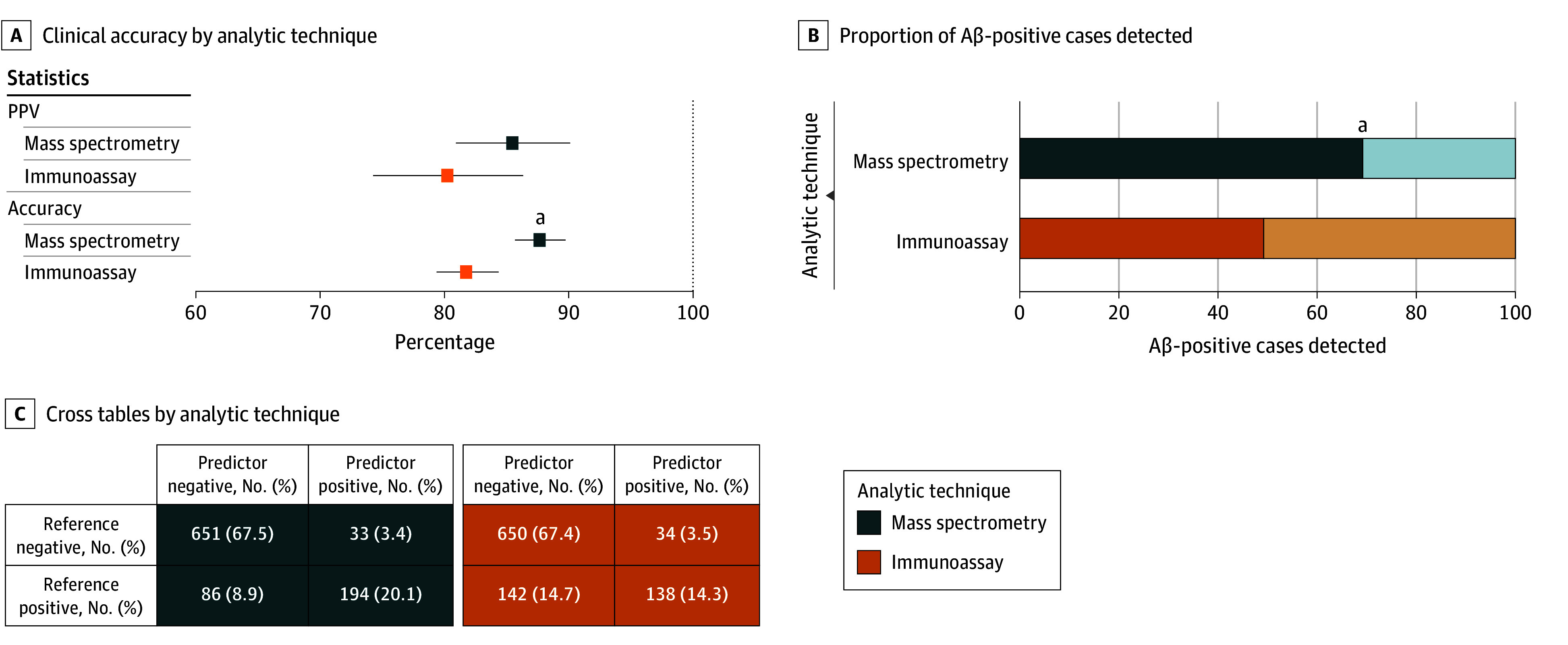
Comparison of Plasma Only as a Confirmatory Test for Prediction of Amyloid Positivity Using Mass Spectrometry vs Highly Accurate Immunoassay Clinical accuracy of plasma phosphorylated tau 217 measured by mass spectrometry and immunoassay is compared for assessing amyloid β (Aβ) positivity based on either cerebrospinal fluid (CSF) or positron emission tomography (PET), using thresholds set at 95% specificity in the whole sample. Dashed lines indicate the maximum values achievable by a perfect biomarker. The percentage of positive individuals selected by plasma is shaded in the right column. In the bar plots, individuals who would not be selected out of those who were Aβ positive (ie, false-negative rate) are shown in a lighter color. Mass spectrometry methods were all performed at Washington University or by C2N Diagnostics, independently for each cohort. Immunoassay methods included Eli Lilly and Janssen platforms. PPV indicates positive predictive value. ^a^Statistically significant differences (*P* < .05) in the specified statistic when comparing immunoassay to mass spectrometry.

Plasma ratios of p-tau217 to non–p-tau217 (termed %p-tau217) as measured by mass spectrometry have been shown to be superior to immunoassay-based p-tau217 tests^12^ and noninferior to US Food and Drug Administration–approved CSF AD tests.^18^ However, immunoassay-based methods require less complex infrastructure and can be automated, streamlining their use. Therefore, we performed a head-to-head comparison in all the participants with %p-tau217 measured mass spectrometry vs p-tau217 measured by immunoassay (n = 964, eTable 8 in Supplement 1). Notably, both methods were measured with different assays for different cohorts, so we *z*-transformed all p-tau217 levels. The difference between Aβ-positive vs Aβ-negative cases in 964 individuals was larger in mass spectrometry–based %p-tau217 than for immunoassay-based p-tau217 (Cohen *d*, 1.47 vs Cohen *d*, 1.29, respectively; *P* < .001) (eFigure 2C-E in Supplement 1). The mass spectrometry method also had significantly higher overall accuracy (88% [95% CI, 86-90] vs 82% [95% CI, 79-84]; *P* < .001) and true Aβ-positive detection rate (69% [95% CI, 64-75] vs 49% [95% CI, 43-55]; *P* < .001), while PPVs were not significantly different (85% [95% CI, 81-90] vs 80% [95% CI, 74-86]; *P* = .12) (Figure 4; eTable 9 in Supplement 1). Similar results were obtained when studying BioFINDER-2 only individuals, in which only 1 method was used for immunoassay and 1 for mass spectrometry (eTable 10 in Supplement 1).

### Sensitivity Analyses

Additional analyses incorporating both age and *APOE* ε4 carriership showed significantly improved accuracy (81% vs 83%) and detection rates for Aβ-positive cases (46% vs 51%) (eFigure 5 in Supplement 1), compared to using age alone. Findings using alternative Aβ PET positivity thresholds are presented in eFigure 6 in Supplement 1. A lower threshold (more sensitive; Centiloid >12) increased PPVs but reduced overall accuracy and detection rates, while a higher threshold (more specific; Centiloid >37) had the opposite effect.

In the 2-step approach, we applied a more sensitive plasma p-tau217 threshold (85% specificity) only when followed by a CSF or PET test. The rationale was that less specific tests are suitable for screening, as more specific tests can later confirm the presence of pathology. This strategy increased the proportion of positive cases (55%-81% for 85% specificity vs 44%-59% for 95% specificity threshold) while maintaining high PPVs (81%-97% vs 91%-99%) (eFigure 7 in Supplement 1). Similar to our previous analysis, using a more sensitive plasma p-tau217 threshold increased the number of CSF and PET tests performed (−57% for 75% specificity vs −75% for 97.5% specificity threshold, compared to using them with all individuals) and the final number of participants included in the trial (30% vs 6%) (eFigure 8 in Supplement 1). Also, less participants required plasma screening (17% for 75% specificity vs 131% for 97.5% specificity threshold), but lower percentage of plasma-positive cases were subsequently confirmed by CSF or PET tests (61% vs 87%) (eFigure 8 in Supplement 1).

Sensitivity analyses restricted to plasma p-tau217 measurements from a single assay and site (Eli Lilly, measured in Lund University; n = 1670) showed improved accuracy (85% vs 81%) and detection rates (56% vs 46%), although PPVs remained similar (80% vs 79%) (eTable 11 in Supplement 1). PPVs also increased with age (<60 years: 38% to ≥80 years: 93%), while NPVs decreased with age (<60 years: 91% to ≥80 years: 66%), when using plasma p-tau217 levels as predictor of Aβ positivity (eTable 12 in Supplement 1).

## Discussion

The main finding of this cohort study is that plasma p-tau217 was an effective marker for identifying Aβ positivity in cognitively unimpaired individuals. When used alone, plasma p-tau217 correctly identified approximately 80% of Aβ-positive cases. A 2-step approach, involving confirmatory CSF or PET testing following a positive plasma p-tau217 result, enhanced the PPV to greater than 95%, while maintaining high sensitivity. This strategy reduced the need for PET or CSF testing by over 40%, compared with a workflow that omits plasma screening. Compared to immunoassay methods, a mass spectrometry–based p-tau217 test, which might be less scalable, achieved somewhat higher accuracy and detected more Aβ-positive individuals, but PPVs were comparable between methods. These findings underscore the clinical value of plasma p-tau217 as a tool for early AD detection, with implications for secondary prevention trials and future treatment programs when available.

From a clinical perspective, plasma p-tau217, in combination with brief cognitive assessment, may serve as an effective screening tool in primary and secondary care, particularly in clinical settings where access to PET imaging or lumbar puncture is limited. Integration into clinical practice is currently constrained by the lack of approved treatments for asymptomatic individuals. However, this landscape is expected to change significantly as disease-targeting treatments become available for preclinical AD. In anticipation of this shift, our findings have direct ramifications for the design of preclinical AD trials, where minimizing the inclusion of Aβ-negative participants is essential. The high PPVs observed in our study with plasma p-tau217 are particularly relevant in these trial settings, whereas a lower NPV—and the associated risk of missed Aβ-positive cases—is less detrimental. Although PPVs in cognitively unimpaired individuals were slightly lower than those reported in symptomatic populations (ie, mild cognitive impairment or dementia),^18^ this difference mainly reflects the lower Aβ prevalence in cognitively unimpaired individuals, which is a key determinant of PPV.^41^ To improve detection performance, we evaluated more sensitive p-tau217 quantification methods. Consistent with prior work,^12^ mass spectrometry improved sensitivity by identifying additional Aβ-positive cases, although PPVs remained comparable to those achieved with immunoassays. This highlights the need for complementary strategies to reach higher certainty in plasma-positive cases.

In this regard, implementing a 2-step approach^42^ combining plasma screening with confirmatory CSF or PET testing proved effective in reducing false positives without compromising sensitivity. This approach may substantially reduce cost and patient burden relative to testing all individuals with CSF or PET. In contrast, relying solely on plasma p-tau217 without confirmatory CSF or PET to achieve a PPV over 90% would require more stringent thresholds, which in turn would reduce sensitivity. While ongoing efforts to standardize plasma collection and analysis may improve sensitivity, this trade-off is especially relevant in trials relying solely on plasma p-tau217 to detect preclinical AD, such as TRAILBLAZER-ALZ3 (NCT05026866) and TRAILRUNNER-ALZ3 (NCT06653153), and in potential future clinical applications. The 2-step alternative strategy, as adopted in studies like AHEAD 3-45,^38^ preserves high enrollment rates while mitigating false positive risks. Ultimately, strategy selection should align with the intervention’s risk profile: a 1-step approach may suffice for low-risk, cost-effective treatments, whereas higher-risk or resource-intensive therapies may warrant a 2-step approach to optimize PPV. Tailoring plasma thresholds and selection strategies to specific clinical or trial objectives is thus essential.

Our analyses also displayed differential performance between CSF and PET reference standards, likely due to their detection of different phases of Aβ pathology. CSF Aβ biomarkers tend to become abnormal earlier than Aβ PET.^43^ Accordingly, CSF identified more true positives, while Aβ PET exhibited a higher PPV. To better align PET classification with CSF detection in early-stage populations,^36^ we applied a relatively sensitive quantitative Aβ-PET threshold (25 Centiloids) while being clinically relevant, consistent with prior studies^35^ and supported by our own data (eFigure 1 in Supplement 1).^27^ This threshold approximates traditional visual-read criteria for Aβ positivity.^37^ Sensitivity analyses confirmed that more stringent PET thresholds improved accuracy but reduced PPV, reflecting the impact of disease prevalence on predictive metrics.

### Strengths and Limitations

Previous studies in cognitively unimpaired populations have often been limited by small sample sizes,^46^ with notable exceptions like the A4 and LEARN studies.^48^ Our study, drawing from 12 independent cohorts, strengthens the generalizability of these findings and underscores the importance of large-scale, population-representative plasma biomarker studies in early AD. As the field moves toward earlier detection and intervention, cognitively unimpaired individuals will play an increasingly central role in clinical trials and therapeutic strategies.^7^ Thus, our results can help guide the design of future trials and treatment protocols. Nonetheless, we acknowledge several limitations. The use of multiple cohorts introduced variability in biomarker measurements. To mitigate this, we used harmonization methods, such as the Centiloid scale^34^ for PET, or using Aβ42 ratios with p-tau181 or Aβ40 for CSF to account for variability in production and clearance rates^49^ and applying cohort-specific thresholds consistent with clinical practices. While plasma p-tau217 levels were harmonized using cohort-specific *z* scores, a unified protocol, such as the CentiMarker,^50^ may further enhance reproducibility. Further work should be done for the establishment of reliable and generalizable cutoffs for plasma p-tau217. Additionally, the Aβ prevalence in our sample was slightly higher than in the general cognitively unimpaired population, suggesting the need for validation in more diverse samples.

## Conclusions

The findings in this study support the clinical utility of plasma p-tau217 as a stand-alone tool for identifying preclinical AD but suggest that confirmatory CSF or PET test in those with abnormal p-tau217 would further improve the PPV in many clinical scenarios. The latter 2-step approach may streamline accurate identification of individuals with preclinical AD. The use of plasma p-tau217 as a stand-alone tool or as part of a 2-step approach will reduce resource demands, and minimize unnecessary burdensome PET or CSF procedures, ultimately accelerating the development and implementation of early-stage AD therapeutics.

## Notes

Supplement 1. eFigure 1. Statistics by different thresholds eFigure 2. Associations between plasma p-tau217 Aβ-status across methods eFigure 3. Characteristics of participants selected by the two-step, by plasma-only or by invasive tests-only approaches eFigure 4. Saved costs in clinical trial selection by approach eFigure 5. Plasma p-tau217 clinical accuracy with and without covariates eFigure 6. Sensitivity analyses with different Aβ-PET quantification thresholds eFigure 7. Comparison of clinical accuracy between two-step approach and plasma -only or invasive tests-only when using a lower specificity threshold eFigure 8. Implications of two-step approach for clinical trials using different plasma thresholds eTable 1. Cohorts description eTable 2. Description of plasma methods by cohort eTable 3. Description of Aβ-PET methods by cohort eTable 4. Description of CSF methods by cohort eTable 5. Sample characteristics by cohort eTable 6. Statistics of plasma p-tau217 as stand-alone confirmatory marker of Aβ-positivity eTable 7. Statistics for the two-step approach, plasma-only, and invasive test-only strategies eTable 8. Sample characteristics of participants with both immuno-assay and mass-spectrometry based plasma data eTable 9. Statistics for the different plasma assays as stand-alone confirmatory marker of Aβ-positivity eTable 10. Statistics of plasma p-tau217 as stand-alone confirmatory marker of Aβ-positivity for a subsample with same p-tau217 assay eTable 11. Statistics for the different plasma as stand-alone confirmatory marker of Aβ-positivity for different age ranges

Supplement 2. ADNI, ALFA, and PREVENT-AD Study Groups

Supplement 3. Data sharing statement
