# Supplementary material for: Plasma Phosphorylated Tau 217 to Identify Preclinical Alzheimer Disease
Source: JAMA Neurol. 2025 Sep 15:e253217. Online ahead of print. doi: 10.1001/jamaneurol.2025.3217 (PMC12558403; doi:10.1001/jamaneurol.2025.3217)
Supplement: Supplement 1. — eFigure 1. Statistics by different thresholds eFigure 2. Associations between plasma p-tau217 Aβ-status across methods eFigure 3. Characteristics of participants selected by the two-step, by plasma-only or by invasive tests-only approaches eFigure 4. Saved costs in clinical trial selection by approach eFigure 5. Plasma p-tau217 clinical accuracy with and without covariates eFigure 6. Sensitivity analyses with different Aβ-PET quantification thresholds eFigure 7. Comparison of clinical accuracy between two-step approach and plasma -only or invasive tests-only when using a lower specificity threshold eFigure 8. Implications of two-step approach for clinical trials using different plasma thresholds eTable 1. Cohorts description eTable 2. Description of plasma methods by cohort eTable 3. Description of Aβ-PET methods by cohort eTable 4. Description of CSF methods by cohort eTable 5. Sample characteristics by cohort eTable 6. Statistics of plasma p-tau217 as stand-alone confirmatory marker of Aβ-positivity eTable 7. Statistics for the two-step approach, plasma-only, and invasive test-only strategies eTable 8. Sample characteristics of participants with both immuno-assay and mass-spectrometry based plasma data eTable 9. Statistics for the different plasma assays as stand-alone confirmatory marker of Aβ-positivity eTable 10. Statistics of plasma p-tau217 as stand-alone confirmatory marker of Aβ-positivity for a subsample with same p-tau217 assay eTable 11. Statistics for the different plasma as stand-alone confirmatory marker of Aβ-positivity for different age ranges [file jamaneurol-e253217-s001.pdf]

# Supplemental Online Content

Salvadó G, Janelidze S, Bali D, et al; ADNI Study Group; ALFA Study Group; PREVENT-AD Study Group. Plasma phosphorylated tau 217 to identify preclinical Alzheimer disease. *JAMA Neurol*. Published online September 15, 2025. doi:10.1001/jamaneurol.2025.3217

**eFigure 1.** Statistics by different thresholds
**eFigure 2.** Associations between plasma p-tau217 Aβ-status across methods
**eFigure 3.** Characteristics of participants selected by the two-step, by plasma-only or by invasive tests-only approaches
**eFigure 4.** Saved costs in clinical trial selection by approach
**eFigure 5.** Plasma p-tau217 clinical accuracy with and without covariates
**eFigure 6.** Sensitivity analyses with different Aβ-PET quantification thresholds
**eFigure 7.** Comparison of clinical accuracy between two-step approach and plasma -only or invasive tests-only when using a lower specificity threshold
**eFigure 8.** Implications of two-step approach for clinical trials using different plasma thresholds
**eTable 1.** Cohorts description
**eTable 2.** Description of plasma methods by cohort
**eTable 3.** Description of Aβ-PET methods by cohort
**eTable 4.** Description of CSF methods by cohort
**eTable 5.** Sample characteristics by cohort
**eTable 6.** Statistics of plasma p-tau217 as stand-alone confirmatory marker of Aβ-positivity
**eTable 7.** Statistics for the two-step approach, plasma-only, and invasive test-only strategies
**eTable 8.** Sample characteristics of participants with both immuno-assay and mass-spectrometry based plasma data
**eTable 9.** Statistics for the different plasma assays as stand-alone confirmatory marker of Aβ-positivity
**eTable 10.** Statistics of plasma p-tau217 as stand-alone confirmatory marker of Aβ-positivity for a subsample with same p-tau217 assay
**eTable 11.** Statistics for the different plasma as stand-alone confirmatory marker of Aβ-positivity for different age ranges

This supplemental material has been provided by the authors to give readers additional information about their work.

© 2025 Salvadó G et al. *JAMA Neurology*.

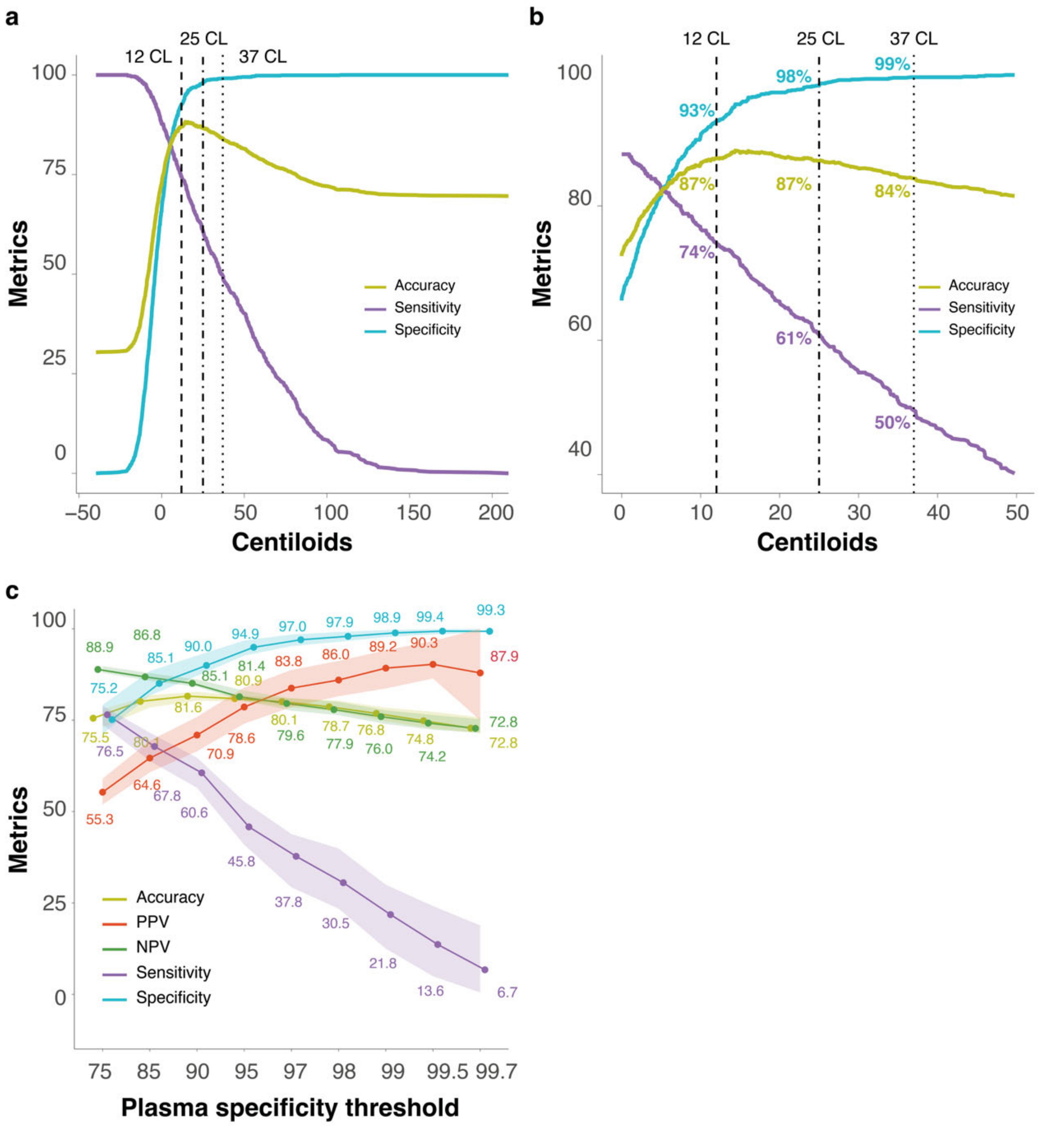


**eFigure 1: Statistics by different thresholds**

Overall accuracy, sensitivity, and specificity for different Centiloid thresholds against AD CSF biomarkers for in all individuals with Centiloid quantification and CSF biomarkers (n=1,652) are shown in the first row (a-b). (b) represents a zoomed-in version of the data between 0-50 Centiloids. Vertical lines represent the 12, 25, and 37 Centiloid thresholds. Metrics of plasma-only approach for different specificity thresholds are shown in c. In c, x-axis has same distance separation for all thresholds. Statistics in b and c are shown in the figure color-coded.

© 2025 Salvadó G et al. *JAMA Neurology*.

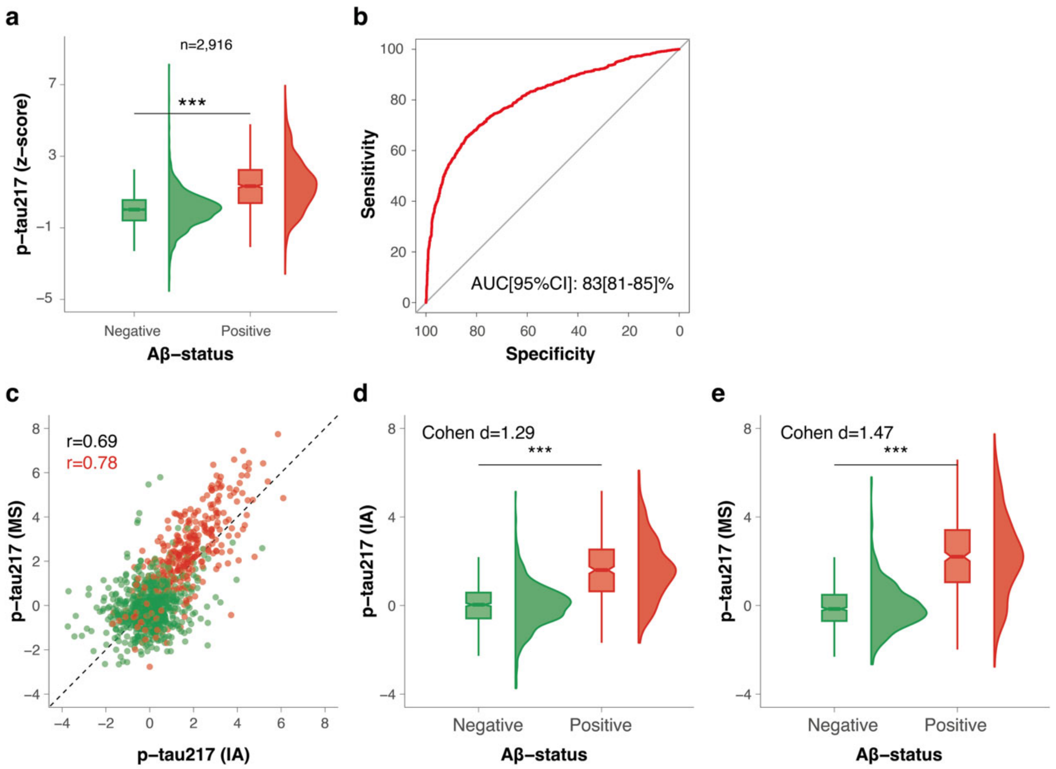


**eFigure 2: Associations between plasma p-tau217 Aβ-status across methods**

(a) Plasma p-tau217 levels, measured using immunoassay (IA) methods, are shown by Aβ-status, with either CSF or PET as the reference standard. (b) Area under the curve (AUC) for plasma p-tau217 (IA) and age in relation to Aβ-status assessment. (c) Correlation between plasma p-tau217 levels measured by mass-spectrometry (MS) and IA across all participants with available data. Pearson's correlation values are shown for all participants (black) and Aβ-positive participants only (red) in the top-left corner. The dashed line represents identity. (d-e) Differences in plasma p-tau217 levels measured by immunoassay (d) or mass-spectrometry (e) based on Aβ-status. ***: $p < 0.001$.

© 2025 Salvadó G et al. *JAMA Neurology*.

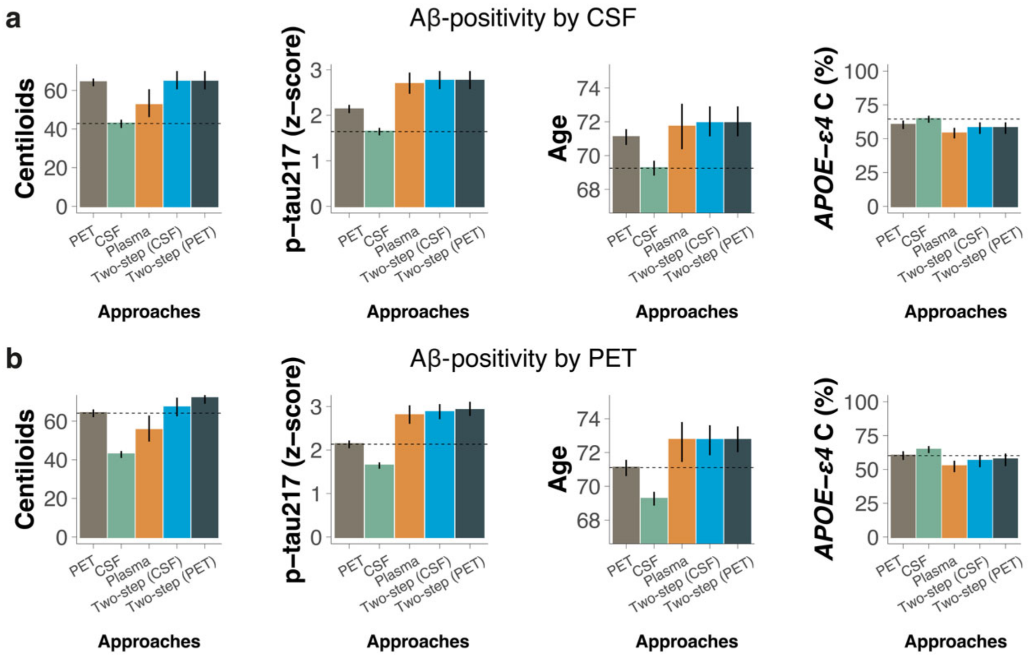


**eFigure 3: Characteristics of participants selected by the two-step, by plasma-only or by invasive tests-only approaches**

Characteristics of Aβ-load, plasma p-tau217 levels, and demographic factors (age and APOE-ε4 carriership) are shown for participants selected by each approach, with CSF (a) or PET (b) used as the reference standard. Results for CSF-only (a) and PET-only (b) approaches are included for comparison. Dashed horizontal lines represent the mean value for each category based on the reference standard. Plasma positivity was determined using a threshold based on 95% specificity for all approaches.

© 2025 Salvadó G et al. *JAMA Neurology*.

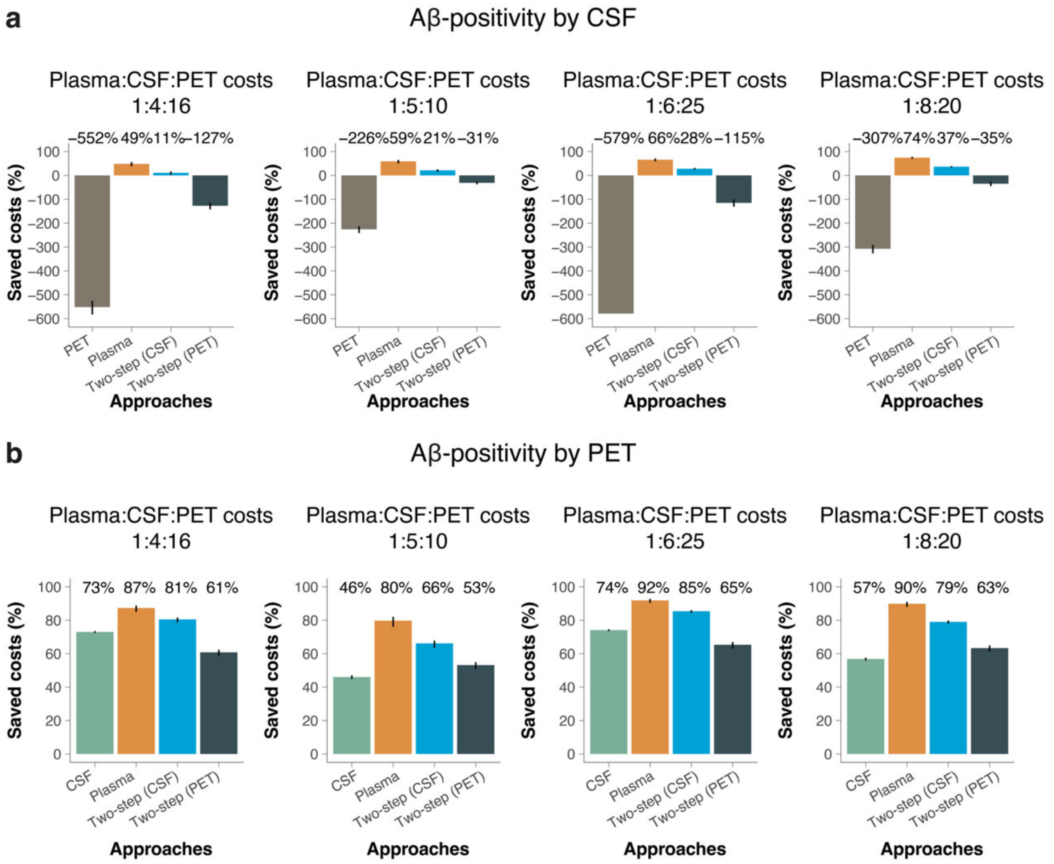


**eFigure 4: Saved costs in clinical trial selection by approach**

This figure illustrates the saved costs of a hypothetical clinical trial aiming to recruit 100 Aβ-positive participants, comparing different confirmatory approaches and cost ratios, using either CSF (a) or PET (b) as the reference standard. The investigated approaches include: an Aβ-PET-only approach (dark blue), a CSF-only approach (light blue), a plasma-only approach (red), a two-step approach using plasma followed by CSF (light green), and a two-step approach with PET as the second step (dark green). The costs are compared to those associated with using a CSF-only (a) or PET-only (b) approach. Plasma p-tau217 was assessed as positive using a 95% specificity threshold in all cases.

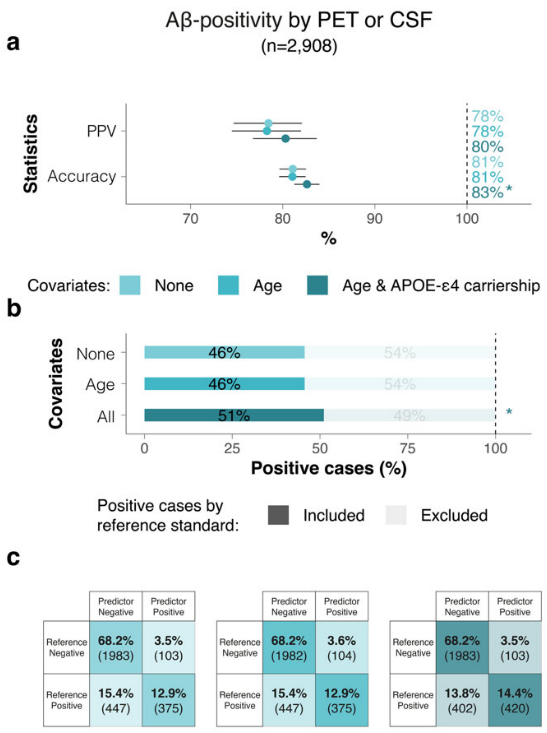


**eFigure 5: Plasma p-tau217 clinical accuracy with and without covariates**

This figure shows the clinical accuracy of the plasma-only approach, comparing results without covariates (lighter blue), with adjustment for age only (mid blue), and with adjustment for both age and *APOE-ε4* carriership (dark blue), against Aβ-positivity assessed by either CSF or Aβ-PET. The percentage of positive (negative) cases, based on the reference standard, for the positive cases selected by plasma, are shaded on the right column. Dashed lines represent the maximum values obtainable by a perfect biomarker. Cross-tables comparing predicted versus reference status for each outcome are shown in panel b for all three approaches (color-coded).

© 2025 Salvadó G et al. *JAMA Neurology*.

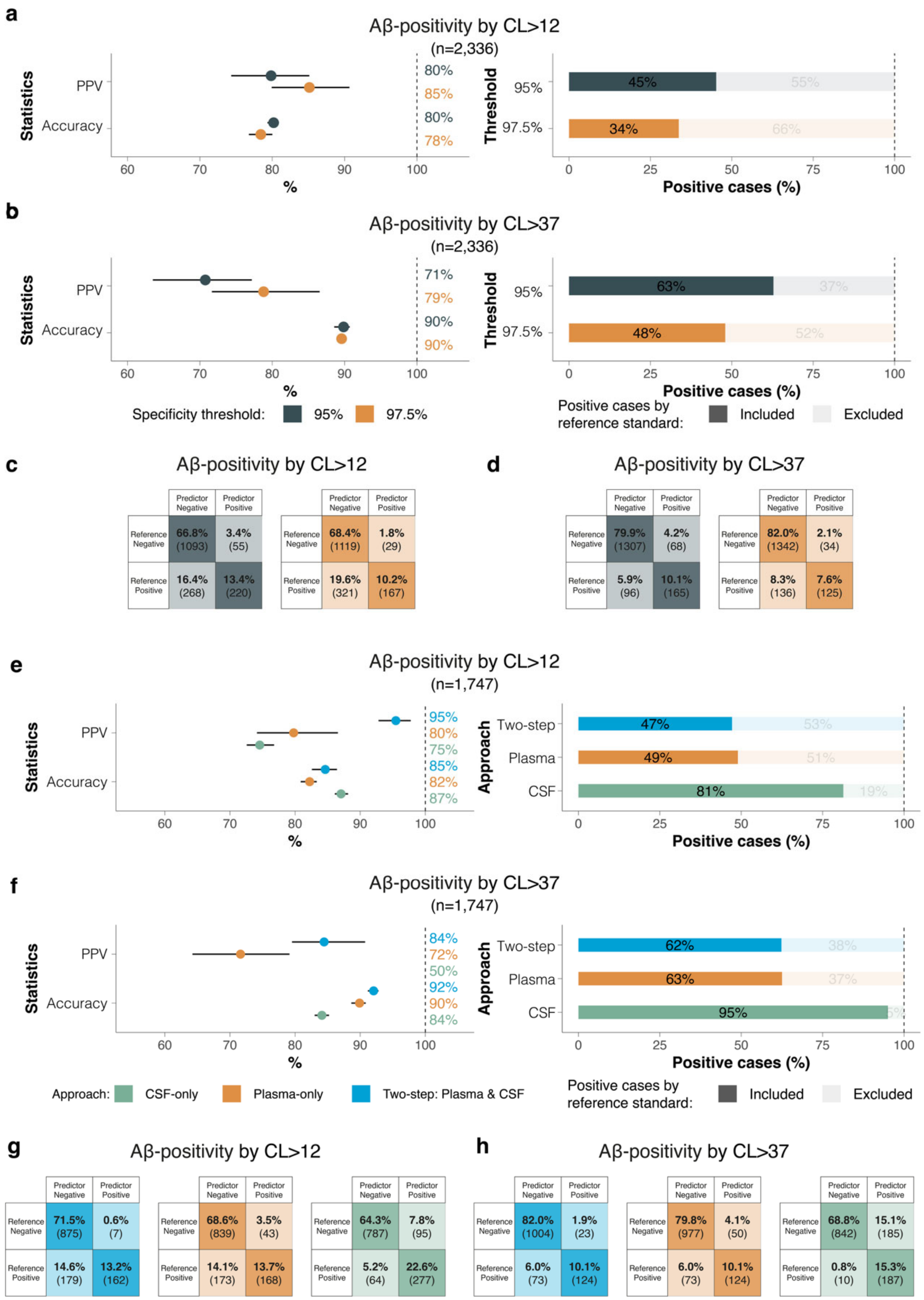


**eFigure 6: Sensitivity analyses with different Aβ-PET quantification thresholds**

Panels a-d present the clinical accuracy of the plasma-only approach and panels e-h present the clinical accuracy between the two-step approach, plasma-only, and invasive test-only strategies, when using different thresholds of Aβ-PET quantification as the standard of truth (12 Centiloids or 37 Centiloids). Dashed lines represent the maximum values attainable by a perfect biomarker. (a-b, e-f). The percentage

© 2025 Salvadó G et al. *JAMA Neurology*.

of positive (negative) cases, based on the reference standard, for the positive cases selected by plasma, are shaded on the right column (a-b, e-f). Cross-tables comparing predicted versus reference status for each outcome are shown in panels c-d for both specificity thresholds (color-coded) and in g-h for all approaches (color-coded). Only participants with Centiloid quantification (a-d: n=2,336; e-h: n=1,747) are included in these analyses.

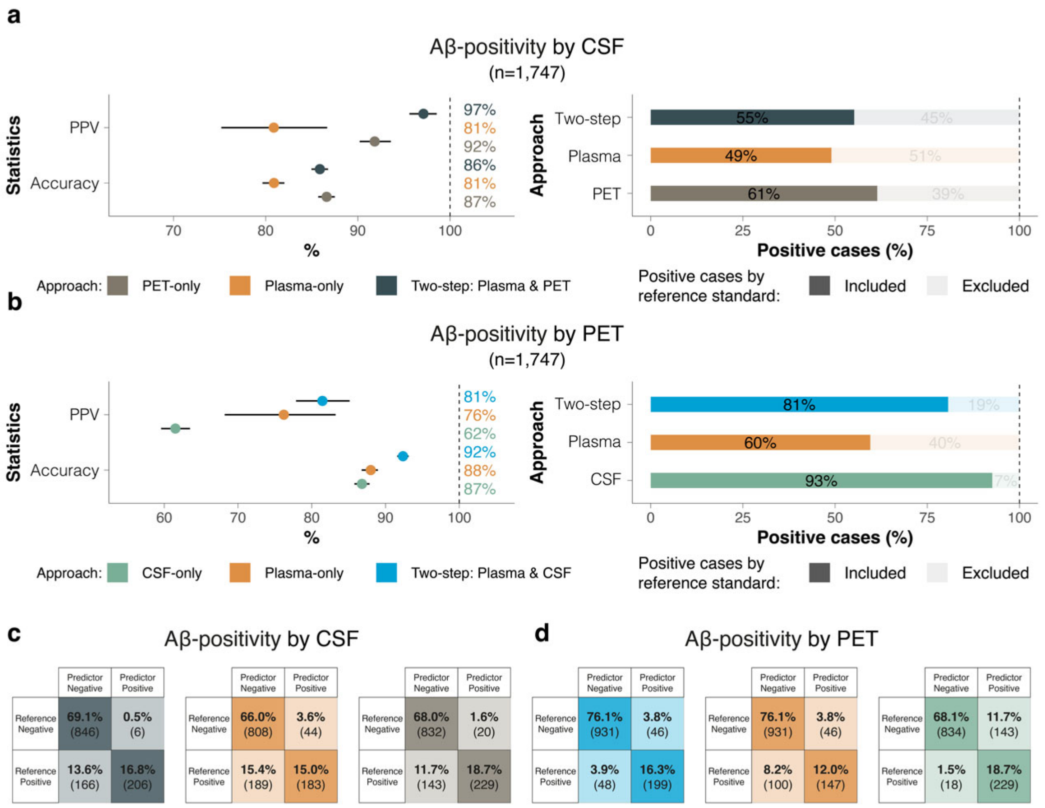


**eFigure 7: Comparison of clinical accuracy between two-step approach and plasma -only or invasive tests-only when using a lower specificity threshold**

Plasma positivity was determined using a threshold based on 95% specificity for the plasma-only approach, while a more liberal threshold (85% specificity) was applied in the two-step approach. Results are shown for using CSF (a) or Aβ-PET (b) as the reference standard. The percentage of positive (or negative) cases by the reference standard among positive cases selected by plasma is shaded on the right column. Dashed lines represent the maximum values attainable by a perfect biomarker. (d-e) Cross-tables illustrate predicted vs. reference status for each outcome, comparing the three approaches (indicated by color).

© 2025 Salvadó G et al. *JAMA Neurology*.

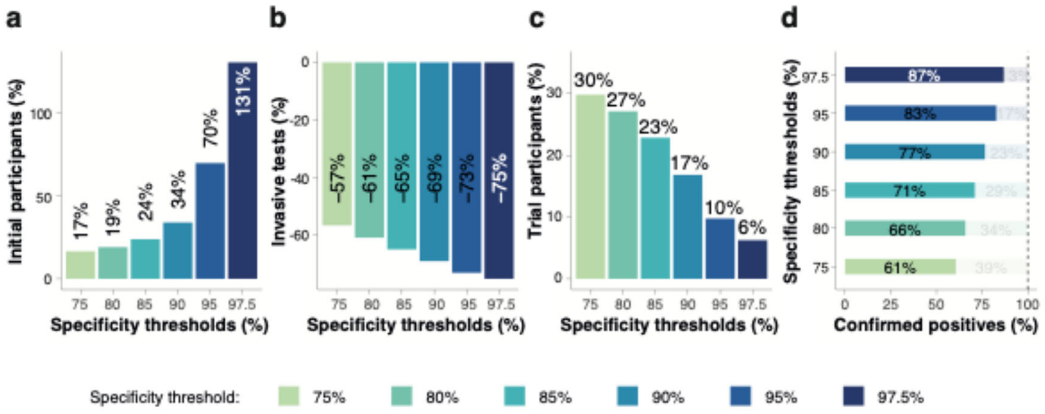


**eFigure 8: Implications of two-step approach for clinical trials using different plasma thresholds**

This figure shows the implications of using varying plasma thresholds (from liberal to conservative, represented by light green to dark blue) in the two-step approach, where plasma is assessed first and followed by CSF, using PET as the reference standard. Lighter portions of the bar plots in the third column (c) represent the false Aβ-positive participants that would be included in the hypothetical trial. he percentage of positive (or negative) cases by the reference standard for the positive cases selected by plasma is shaded in the right column.

| Cohort | Cohort description | References |
|---|---|---|
| **ADC** | The Amsterdam Dementia Cohort (ADC) is a prospective cohort study including (amongst others) individuals with subjective cognitive decline (SCD) presenting at the Alzheimer Center of the VU University Medical Center Amsterdam. All participants have been referred to the memory clinic by their general practitioner, and a neurologist or geriatrician in the case of a second opinion for evaluation of cognitive complaints. They receive standardized dementia screening at the memory clinic, including an interview with a neurologist, physical and neurological examination, neuropsychological assessment. Individuals with SCD can additionally be included in the SCIENCe study, for which the main inclusion criteria are a diagnosis of SCD (i.e., cognitive complaints and normal cognition) and age ≥ 45 years. Exclusion criteria for participation in the SCIENCe study are MCI, dementia, major psychiatric disorder (i.e., current depression, personality disorders, schizophrenia), neurological diseases known to cause memory complaints (i.e., Parkinson's disease, epilepsy), HIV, abuse of alcohol or other substances, and language barrier. | [1] |
| **ADNI** | The ADNI study was launched in 2003 as a public-private partnership, led by Principal Investigator Michael W. Weiner, MD. The primary goal of ADNI has been to test whether MRI, PET, other biological markers, and clinical and neuropsychological assessment can be combined to measure the progression of mild cognitive impairment (MCI) and early AD. Participants, typically aged 55 to 90, must meet specific health criteria, including the absence of other significant neurological conditions, psychiatric disorders, or unstable medical conditions that could affect study outcomes. Participants undergo regular neuroimaging, cognitive | [2] |

© 2025 Salvadó G et al. *JAMA Neurology*.

| | | |
|---|---|---|
| | testing, and biomarker analysis, including blood and cerebrospinal fluid samples. Optional genetic testing, such as APOE genotyping, is also conducted. | |
| **AIBL** | The Australian Imaging, Biomarker & Lifestyle Flagship Study of Ageing (AIBL) is a longitudinal, prospective cohortwith participants coming from two-site study – Melbourne and Perth. To be included in the study, participants were (1) ≥60 years old; (2) fluent in English; (4) had completed at least 7 years of education; (5) did not have any history of neurological or psychiatric disorders, drug or alcohol abuse or dependence, or any other unstable medical condition; and (6) were deemed to be cognitively unimpaired (CU), based on their performance on a battery of cognitive assessments that AIBL participants undergo every 12 to 18 months. A multidisciplinary clinical review panel determines whether an individual is CU, based on the available clinical and neuropsychological information. | [3] |
| **ALFA+** | The ALFA parent cohort comprises cognitively normal participants aged 45 to 74, who undergo cognitive tests, provide clinical history, lifestyle information, and blood samples for genetic analysis at the Barcelonaβeta Brain Research Center (BBRC). This cohort serves as the foundation for establishing research protocols and conducting both observational and interventional studies on preclinical participants at risk of cognitive impairment due to AD. The ALFA+ study involves more detailed phenotyping, including similar characterizations as in the ALFA parent cohort, along with the collection of additional biomarkers through CSF, blood, and urine samples, as well as imaging biomarkers via MRI and PET. | [4] |
| **BioFINDER-1 & BioFINDER-2** | The Swedish BioFINDER studies are longitudinal studies covering the entire AD continuum in which participants were recruited at Skåne University Hospital and the Hospital of Ängelholm, Sweden. The main inclusion criteria were absence of cognitive symptoms as assessed by a physician with special interest in | [5] |

© 2025 Salvadó G et al. *JAMA Neurology*.

| | | |
|---|---|---|
| | cognitive disorders, being fluent in Swedish, having no significant unstable systemic illness that made it difficult to participate in the study, having no current significant alcohol or substance misuse, and no significant neurological or psychiatric illness. For the current study participants above > 50 years old were included. Both cognitively healthy older adults and SCD participants were included. The SCD participants were referred from participating memory clinic because of cognitive complaints, but did not fulfill criteria for MCI (defined using criteria by Petersen and operationalized according to1,2) following a neuropsychological test battery. | |
| **Knight ADRC** | The Charles F. and Joanne Knight Alzheimer Disease Research Center (Knight ADRC) is one of approximately 30 Centers funded by the National Institute on Aging (NIA) located at major medical institutions across the United States. Researchers at these Centers are working to translate research advances into improved diagnosis and care for people with Alzheimer disease, as well as working to find a treatment or way to prevent Alzheimer disease and other types of dementia. | [6] |
| **MCSA** | The Mayo Clinic Study of Aging (MCSA) is a longitudinal population-based study of cognitive aging in Olmsted County, Minnesota. The study was designed to study prevalence, incidence and risk factors for MCI and dementia. Potential participants are randomly enumerated from the Olmsted County, MN, census and enrolled by age/sex strata. Enumeration is repeated to maintain a sample of approximately 3000 active participants. At entry, every person underwent evaluations that included a medical history review and interview with the participant and a study partner, a neurological examination by a physician; and a neuropsychological examination. For this study, participants were considered MCI only if the study coordinator, physician, and neuropsychologist were all in agreement regarding the MCI diagnosis. | [7] |

© 2025 Salvadó G et al. *JAMA Neurology*.

| | | |
|---|---|---|
| | Participants were judged cognitively normal if they did not meet MCI criteria. Participants aged between 50 and 89 years old were included in the current study. | |
| **PREVENT-AD** | The PREVENT-AD (Pre-symptomatic Evaluation of Experimental or Novel Treatments Alzheimer Disease) cohort is composed of cognitively healthy participants over 55 years old, at risk of developing Alzheimer Disease (AD) as their parents and/or siblings were/are affected by the disease. These 'at-risk' participants have been followed for a naturalistic study of the presymptomatic phase of AD since 2011 using multimodal measurements of various disease indicators. Two clinical trials intended to test pharmaco-preventive agents have also been conducted. | 8 |
| **SPIN** | The SPIN cohort is a comprehensive observational platform for studying neurodegenerative diseases that uses multiple types of biomarkers and takes an integrative approach. Individuals who participate in SPIN agree to donate biofluid (blood and CSF) and undergo detailed neurological and neuropsychological evaluations. Participants are followed for at least 4 years, with additional samples and imaging studies taken every other year. | 9 |
| **TRIAD** | The Translational Biomarkers of Aging and Dementia (TRIAD) cohort study is a longitudinal observational cohort study in Montréal, Québec, Canada. Participants are recruited from the community and from the the McGill Centre for Studies in Aging. All participants are clinically evaluated by dementia specialists. Participants were excluded from this study if they had systemic conditions which were not adequately controlled through a stable medication regimen. Other exclusion criteria were active substance abuse, recent head trauma, recent major surgery, or MRI/PET safety contraindications. The study was approved by the | 10 |

© 2025 Salvadó G et al. *JAMA Neurology*.

| | | |
|---|---|---|
| | Montreal Neurological Institute PET working committee and the Douglas Mental Health University Institute Research Ethics Board. Written informed consent was obtained for all participants. | |
| **WRAP** | The Wisconsin Registry for Alzheimer's Prevention is a longitudinal observational cohort study enriched with persons with a parental history (PH) of probable Alzheimer's disease (AD) dementia. Recruitment sources included memory clinics in which a parent was diagnosed or treated, limited radio and newspaper advertisements, and word of mouth. Participants generally meet the following inclusion criteria at study entry: age 40–65 years; fluent English speaker; visual and auditory acuity adequate for neuropsychological testing; good health with no diseases expected to interfere with study participation over time. Participants are excluded from enrollment if they have a prior diagnosis of dementia or evidence of dementia at baseline testing (one was excluded due to baseline dementia). | [11] |

**eTable 1: Cohorts description**

© 2025 Salvadó G et al. *JAMA Neurology*.

| Cohort | Plasma assay | Methods | References |
|---|---|---|---|
| **ADC** | Lilly | Plasma p-tau217 levels were measured using an immunoassay developed by Lilly Research Laboratories (IN, USA) on a Meso Scale Discovery platform at Lund University | [1] |
| **ADNI** | Janssen R&D | Plasma p-tau217 levels were measured using Janssen LucentAD Simoa® p-Tau 217 by Quanterix Accelerator Laboratory Services | FNIH_BC_Clinical_Utility_of_Blood_Biomarker_Trajectories_Study_Methodology_Report_20240618.pdf |
| **AIBL** | Janssen R&D | Plasma p-tau217+ levels were measured using an assay developed by Janssen R&D (CA, USA) on a Single Molecule Array (Simoa) HD-X platform | [3] |
| **ALFA+** | Lilly | Plasma p-tau217 levels were measured using in-house immunoassays developed by Eli Lilly and Company, based on the Meso Scale Discovery platform | [12] |
| **BioFINDER-1 & BioFINDER-2** | Lilly | Plasma p-tau217 levels were measured using an immunoassay developed by Lilly Research Laboratories (IN, USA) on a Meso Scale Discovery platform at Lund University | [5] |
| **Knight ADRC** | Lilly | Plasma p-tau217 levels were measured using an immunoassay developed by Lilly Research Laboratories (IN, USA) on a Meso Scale Discovery platform at Lund University | [6] |

© 2025 Salvadó G et al. *JAMA Neurology*.

| | | | |
|---|---|---|---|
| **MCSA** | Lilly | Plasma p-tau217 levels were measured using an immunoassay developed by Lilly Research Laboratories (IN, USA) on a Meso Scale Discovery platform at the Mayo Clinic | 7 |
| **PREVENT-AD** | Lilly | Plasma p-tau217 levels were measured using an immunoassay developed by Lilly Research Laboratories (IN, USA) on a Meso Scale Discovery platform at Lund University | 8 |
| **SPIN** | Lilly | Plasma p-tau217 levels were measured using an immunoassay developed by Lilly Research Laboratories (IN, USA) on a Meso Scale Discovery platform at Lund University | - |
| **TRIAD** | Janssen R&D | Plasma p-tau217 was quantified by scientists at Janssen Research & Development blinded to clinical, demographic and biomarker information. | 13 |
| **WRAP** | Lilly | Plasma p-tau217 levels were measured using an immunoassay developed by Lilly Research Laboratories (IN, USA) on a Meso Scale Discovery platform at Lund University | 11 |

**eTable 2: Description of plasma methods by cohort**

© 2025 Salvadó G et al. *JAMA Neurology*.

| Cohort | Tracer | Scan interval | Methodology | Cut-off | References |
|---|---|---|---|---|---|
| **ADC** | [$^{18}$F]florbetapir / [$^{18}$F]flutemetamol | 0- to 30-min scan and a 90- to 110-min p.i. | Parametric images were generated from SUV ratio (SUVr) and nondisplaceable binding potential ($BP_{ND}$) methods, with cerebellar gray matter as a reference region Visual read following guidelines provided by Avid Radiopharmaceuticals. | NA | 14 |
| **ADNI** | $^{18}$F]florbetapir | 50-70 min p.i. | Global SUVR across cortical summary regions (FS-defined frontal, cingulate, parietal, and lateral temporal) normalized to whole cerebellum. SUVR were converted to Centiloids using previously validated transformations. | >12 CL | ADNI_Centoloid_Methods_Instruction_20210930.pdf |
| **AIBL** | [$^{18}$F]NAV4694 | 50-70 min p.i. | The standard Centiloid (CL) cortical and whole cerebellar volumes of interest template was applied to the summed and spatially normalised PET images in order to obtain SUVR's. These SUVR were transformed into CL units by linear transformation using the PET tracer-specific equations published for conversion of CL method SUVR to CL units | >20CL | 15 |
| **ALFA+** | [$^{18}$F]flutemetamol | 90-110min | PET frames were coregistered. Averaged images were then coregistered to corresponding MRI scans. MRIs were then segmented and normalized to the MNI space together with PET images. SUVr were calculated in MNI space using the target region provided in the GAAIN website (www.gaain.org) and the whole cerebellum as reference region. SUVr values were then transformed to the Centiloid scale. | >25 CL | 16 |

© 2025 Salvadó G et al. *JAMA Neurology*.

| | | | | | |
|---|---|---|---|---|---|
| **BioFINDER-1 & BioFINDER-2** | [$^{18}$F]flutemetamol | 90-110min | Global neocortical composite SUVR for the summed images. with whole cerebellum as reference region | >25 CL | 17 |
| **Knight ADRC** | [$^{11}$C]PIB / [$^{18}$F]florbetapir | Dynamic acquisition /50–70- min p.i. | Data were processed using a region of interest approach using Freesurfer. Amyloid deposition was summarized using the average across the left and right lateral orbitofrontal, medial orbitofrontal, rostral middle frontal, superior frontal, superior temporal, middle temporal, and precuneus regions. | >25 CL | 6 |
| **MCSA** | [$^{11}$C]PIB | 40-60 minutes p.i. | Late uptake amyloid PET images were acquired A meta-ROI was calculated as the voxel-number weighted average of uptake in a target region including prefrontal, orbitofrontal, parietal, temporal, anterior and posterior cingulate, and precuneus regions divided by the uptake in the cerebellar crus gray matter. | >1.48 SUVR (>21CL) | 7 |
| **PREVENT-AD** | [$^{18}$F]NAV4694 | 40 to 70 minutes | Aβ-PET images were realigned onto their respective MRI, masked to remove the scalp and CSF in an attempt to avoid contamination by nongray or nonwhite matter voxels, and smoothed using a full width at half maximum Gaussian kernel of 8mm. Resulting images were scaled using whole cerebellum uptake values (whole cerebellum was preferred to cerebellum gray matter to account better for white matter off-target binding variability between tracers). Global neocortical Aβ burden was quantified by extracting, in native space, the mean standardized uptake value ratio (SUVR) of the frontal, temporal, parietal, and posterior cingulate cortex of the Desikan-Killiany atlas | >25 CL | 8 |

© 2025 Salvadó G et al. *JAMA Neurology*.

| | | | | | |
|---|---|---|---|---|---|
| **TRIAD** | $[^{18}\text{F}]$NAV4694 | 40–70 min p.i. | Scans were reconstructed with the ordered-subset expectation maximization algorithm on a 4-dimensional volume with 3 frames (3 × 600 s) Amyloid-β SUVR from a neocortical region of interest (ROI) for each participant was estimated by averaging the SUVR from the precuneus, prefrontal, orbitofrontal, parietal, temporal, and cingulate cortices, using the cerebellar gray matter as a reference region. | >25 CL | 18 |
| **WRAP** | $[^{11}\text{C}]$PIB | Dynamic acquisition | Amyloid burden was assessed as a global average $^{11}$C-PiB distribution volume ratio (DVR; Logan graphical analysis, cerebellum gray matter reference region), taken across 8 bilateral cortical ROIs. | >1.16 DVR | 19 |

**eTable 3: Description of Aβ-PET methods by cohort**

© 2025 Salvadó G et al. *JAMA Neurology*.

| Cohort | CSF biomarker | CSF assay | Methods | Cut-off | References |
|---|---|---|---|---|---|
| **ADC** | p-tau/Aβ42 | Elecsys | Aβ1– 42, p-tau (181P), and t-tau (Roche Diagnostics GmbH) were analyzed in CSF samples by board-certified technicians using the fully automated Elecsys biomarker assays. | >0.07 | [20] |
| **ADNI** | p-tau/Aβ42 | Elecsys | The Roche Elecsys β-Amyloid(1-42) CSF and Elecsys Phospho-Tau(181P) CSF immunoassays were used following a Roche Study Protocol at the UPenn/ADNI Biomarker Laboratory, | >0.025 | UPENNBIOMK_ROCHE_ELECSYS_METHODS_20231109.pdf |
| **ALFA+** | Aβ42/40 | Elecsys | CSF Aβ40 and Aβ42 were measured with the exploratory NTK robust immunoassays (Roche Diagnostic International Ltd) on a cobas e 411 analyzer or cobas e 601 module at the Clinical Neurochemistry Laboratory, University of Gothenburg, Sweden. | <0.071 | [21] |
| **BioFINDER-1** | Aβ42/40 | Elecsys | CSF Aβ42 and Aβ40 were measured using the Elecsys immunoassays (Roche Diagnostics) | <0.078 | [22] |
| **BioFINDER-2** | Aβ42/40 | Elecsys | CSF Aβ42 and Aβ40 were measured using the Elecsys immunoassays (Roche Diagnostics) | <0.066 | [22] |
| **Knight ADRC** | Aβ42/40 | Lumipulse | CSF Aβ42 and Aβ40 concentrations were measured with an automated immunoassay platform (Lumipulse G1200, Fujirebio) | <0.0673 | [23] |

© 2025 Salvadó G et al. *JAMA Neurology*.

| | | | | | |
|---|---|---|---|---|---|
| **PREVENT-AD** | p-tau/Aβ42 | Innotest | CSF concentrations of the AD biomarkers Aβ1-42 and P181-tau (P-tau) using the Innotest enzyme-linked immunosorbent assay kit (Fujirebio, Ghent, Belgium) | >0.08 | 24 |
| **SPIN** | Aβ42/40 | Lumipulse | Aβ1-42, Aβ1-40, were quantified directly from the storage tubes containing 0.5 mL of CSF using the Lumipulse G β-Amyloid 1-42 and β-Amyloid 1-40 assays on LUMIPULSE G600II automated platform (Fujirebio) and following the manufacturer's instructions. | <0.062 | 25 |
| **TRIAD** | Aβ42/40 | Lumipulse | CSF amyloid-β concentrations (Aβ40 and Aβ42) were measured using the fully automated Lumipulse G1200 instrument (Fujirebio) according to procedures from the manufacturer. | <0.068 | 10 |
| **WRAP** | Aβ42/40 | Elecsys | CSF levels of Aβ42 were measured using the Elecsys β-Amyloid (1-42) electrochemiluminescence immunoassays on a fully automated cobas e 601 instrument (Roche Diagnostics International Ltd., Rotkreuz, Switzerland). CSF Aβ40 levels were measured with robust prototype assays as part of the Roche NeuroToolKit on cobas e 411 and e 601 instruments (Roche Diagnostics International Ltd, Rotkreuz, Switzerland). | <0.047 (batch #1) or <0.056 (batch #2) | 26 |

**eTable 4: Description of CSF methods by cohort**

© 2025 Salvadó G et al. *JAMA Neurology*.

| ADC | | |
|---|---|---|
| | **CSF or PET (N=46)** | **CSF & PET (N=36)** |
| Age, years old | 65.7 (7.87) | 64.5 (6.94) |
| Women, N(%) | 21 (45.7%) | 16 (44.4%) |
| *APOE-ε4* carriers, N(%) | 17 (38.6%) [N=44] | 14 (38.9%) |
| p-tau217, z-score | 1.07 (1.77) | 0.913 (1.75) |
| Aβ-positive, N(%) | 20 (43.5%) | 15 (41.7%) |
| Aβ-PET positive, N(%) | 19 (41.3%) | 14 (38.9%) |
| Centiloids | NA | NA |
| CSF positive, N(%) | 11 (30.6%) [N=36] | 11 (30.6%) |

**eTable 15 Sample characteristics by cohort**

| ADNI | | |
|---|---|---|
| | **CSF or PET (N=241)** | **CSF & PET (N=183)** |
| Age, years old | 71.5 (6.1) | 71.8 (6.2) |
| Women, N(%) | 129 (53.5%) | 97 (53.0%) |
| *APOE-ε4* carriers, N(%) | 73 (30.3%) | 56 (30.6%) |
| p-tau217, z-score | 0.656 (1.34) | 0.652 (1.37) |
| Aβ-positive, N(%) | 89 (36.9%) | 69 (37.7%) |
| Aβ-PET positive, N(%) | 78 (32.4%) | 58 (31.7%) |
| Centiloids | 23.2 (40.2) | 23.2 (40.2) |
| CSF positive, N(%) | 53 (29.0%) [N=183] | 53 (29.0%) |

© 2025 Salvadó G et al. *JAMA Neurology*.

| AIBL | | |
|---|---|---|
| | **CSF or PET (N=180)** | **CSF & PET (N=0)** |
| Age, years old | 74.7 (5.25) | - |
| Women, N(%) | 95 (52.8%) | - |
| *APOE-ε4* carriers, N(%) | 53 (29.4%) | - |
| p-tau217, z-score | 0.205 (1.08) | - |
| Aβ-positive, N(%) | 36 (20.0%) | - |
| Aβ-PET positive, N(%) | 36 (20.0%) | - |
| Centiloids | - | - |
| CSF positive, N(%) | - | - |

| ALFA | | |
|---|---|---|
| | **CSF or PET (N=359)** | **CSF & PET (N=341)** |
| Age, years old | 61.1 (4.66) | 61.1 (4.66) |
| Women, N(%) | 220 (61.3%) | 213 (62.5%) |
| *APOE-ε4* carriers, N(%) | 199 (55.4%) | 185 (54.3%) |
| p-tau217, z-score | 0.251 (1.20) | 0.260 (1.19) |
| Aβ-positive, N(%) | 118 (32.9%) | 116 (34.0%) |
| Aβ-PET positive, N(%) | 34 (9.5%) | 32 (9.4%) |
| Centiloids | 2.83 (17.1) | 2.76 (16.7) |
| CSF positive, N(%) | 116 (34.0%) [N=341] | 116 (34.0%) |

© 2025 Salvadó G et al. *JAMA Neurology*.

| BioFINDER-1 | | |
|---|---|---|
| | **CSF or PET (N=105)** | **CSF & PET (N=104)** |
| Age, years old | 73.5 (5.22) | 73.5 (5.23) |
| Women, N(%) | 69 (65.7%) | 69 (66.3%) |
| *APOE-ε4* carriers, N(%) | 43 (41.0%) | 43 (41.3%) |
| p-tau217, z-score | 0.539 (1.39) | 0.531 (1.40) |
| Aβ-positive, N(%) | 37 (35.2%) | 37 (35.6%) |
| Aβ-PET positive, N(%) | 27 (25.7%) | 27 (26.0%) |
| Centiloids | 22.4 (37.1) | 22.6 (37.2) |
| CSF positive, N(%) | 37 (35.6%) [N=104] | 37 (35.6%) |

| BioFINDER-2 | | |
|---|---|---|
| | **CSF or PET (N=595)** | **CSF & PET (N=592)** |
| Age, years old | 64.3 (11.7) | 64.4 (11.7) |
| Women, N(%) | 312 (52.4%) | 311 (52.5%) |
| *APOE-ε4* carriers, N(%) | 274 (46.1%) | 272 (45.9%) |
| p-tau217, z-score | 0.484 (1.38) | 0.484 (1.38) |
| Aβ-positive, N(%) | 154 (25.9%) | 154 (26.0%) |
| Aβ-PET positive, N(%) | 103 (17.3%) | 103 (17.4%) |
| Centiloids | 5.71 (30.1) | 5.72 (30.2) |
| CSF positive, N(%) | 152 (25.7%) [N=592] | 152 (25.7%) |

© 2025 Salvadó G et al. *JAMA Neurology*.

| Knight ADRC | | |
|---|---|---|
| | **CSF or PET (N=383)** | **CSF & PET (N=234)** |
| Age, years old | 70.2 (6.14) | 69.4 (6.14) |
| Women, N(%) | 223 (58.2%) | 139 (59.4%) |
| *APOE-ε4* carriers, N(%) | 126 (33.1 %) [N=381] | 71 (30.5%) [N=233] |
| p-tau217, z-score | 0.724 (1.63) | 0.754 (1.64) |
| Aβ-positive, N(%) | 119 (31.1%) | 86 (36.8%) |
| Aβ-PET positive, N(%) | 101 (26.4%) | 68 (29.1%) |
| Centiloids | 18.8 (30.8) | 19.9 (33.2) |
| CSF positive, N(%) | 82 (35.0%) [N=234] | 82 (35.0%) |

| MCSA | | |
|---|---|---|
| | **CSF or PET (N=363)** | **CSF & PET (N=0)** |
| Age, years old | 68.3 (12.0) | - |
| Women, N(%) | 166 (45.7%) | - |
| *APOE-ε4* carriers, N(%) | 106 (29.2%) | - |
| p-tau217, z-score | 0.363 (1.21) | - |
| Aβ-positive, N(%) | 108 (29.8%) | - |
| Aβ-PET positive, N(%) | 108 (29.8%) | - |
| Centiloids | - | - |
| CSF positive, N(%) | - | - |

© 2025 Salvadó G et al. *JAMA Neurology*.

| PREVENT-AD | | |
|---|---|---|
| | **CSF or PET (N=217)** | **CSF & PET (N=59)** |
| Age, years old | 69.0 (5.19) | 67.1 (4.59) |
| Women, N(%) | 151 (69.6%) | 42 (71.2%) |
| *APOE-ε4* carriers, N(%) | 84 (38.7%) | 22 (37.3%) |
| p-tau217, z-score | 0.424 (1.38) | 0.300 (1.17) |
| Aβ-positive, N(%) | 49 (22.6%) | 14 (23.7%) |
| Aβ-PET positive, N(%) | 47 (21.7%) | 12 (20.3%) |
| Centiloids | 21.1 (32.7) | 17.3 (24.8) |
| CSF positive, N(%) | 11 (18.6%) [N=59] | 11 (18.6%) |

| SPIN | | |
|---|---|---|
| | **CSF or PET (N=171)** | **CSF & PET (N=0)** |
| Age, years old | 55.5 (12.9) | - |
| Women, N(%) | 123 (71.9%) | - |
| *APOE-ε4* carriers, N(%) | 32 (19.2%) [N=167] | - |
| p-tau217, z-score | 0.174 (1.17) | - |
| Aβ-positive, N(%) | 11 (6.4%) | - |
| Aβ-PET positive, N(%) | - | - |
| Centiloids | - | - |
| CSF positive, N(%) | 11 (6.4%) | - |

© 2025 Salvadó G et al. *JAMA Neurology*.

| TRIAD | | |
|---|---|---|
| | **CSF or PET (N=103)** | **CSF & PET (N=103)** |
| Age, years old | 70.7 (6.39) | 70.7 (6.39) |
| Women, N(%) | 61 (59.2%) | 61 (59.2%) |
| *APOE-ε4* carriers, N(%) | 29 (28.2%) | 29 (28.2%) |
| p-tau217, z-score | 0.326 (1.20) | 0.326 (1.20) |
| Aβ-positive, N(%) | 35 (34.0%) | 35 (34.0%) |
| Aβ-PET positive, N(%) | 25 (24.3%) | 25 (24.3%) |
| Centiloids | 20.0 (29.6) | 20.0 (29.6) |
| CSF positive, N(%) | 35 (34.0%) | 35 (34.0%) |

| WRAP | | |
|---|---|---|
| | **CSF or PET (N=153)** | **CSF & PET (N=95)** |
| Age, years old | 65.8 (6.3) | 65.8 (6.2) |
| Women, N(%) | 97 (63.4%) | 57 (60.0%) |
| *APOE-ε4* carriers, N(%) | 72 (47.1%) | 43 (45.3%) |
| p-tau217, z-score | 0.579 (1.53) | 0.519 (1.58) |
| Aβ-positive, N(%) | 49 (32.0%) | 34 (35.8%) |
| Aβ-PET positive, N(%) | 31 (20.3%) | 16 (16.8%) |
| Centiloids | 8.2 (23.1) | 6.92 (22.8) |
| CSF positive, N(%) | 34 (35.4%) [N=96] | 34 (35.8%) |

© 2025 Salvadó G et al. *JAMA Neurology*.

| Outcome | Threshold | Accuracy | PPV | NPV | Specificity | Sensitivity |
|---|---|---|---|---|---|---|
| Aβ-positivity | 95% | 0.81 [0.80, 0.82] | 0.79 [0.74, 0.84] | 0.82 [0.81, 0.83] | 0.95 [0.93, 0.97] | 0.46 [0.41, 0.53] |
| | 97.5% | 0.80 [0.78, 0.81] | 0.85 [0.80, 0.90] | 0.79 [0.77, 0.81] | 0.97 [0.96, 0.99] | 0.35 [0.27, 0.43] |
| CSF positivity | 95% | 0.82 [0.81, 0.83] | 0.79 [0.74, 0.85] | 0.82 [0.81, 0.85] | 0.95 [0.92, 0.97] | 0.48 [0.41, 0.58] |
| | 97.5% | 0.80 [0.78, 0.81] | 0.85 [0.79, 0.91] | 0.80 [0.77, 0.82] | 0.97 [0.95, 0.99] | 0.36 [0.26, 0.45] |
| Aβ-PET positivity | 95% | 0.86 [0.85, 0.87] | 0.76 [0.70, 0.81] | 0.88 [0.87, 0.89] | 0.95 [0.93, 0.97] | 0.54 [0.49, 0.60] |
| | 97.5% | 0.85 [0.84, 0.86] | 0.82 [0.77, 0.88] | 0.85 [0.84, 0.87] | 0.97 [0.96, 0.99] | 0.41 [0.33, 0.51] |

**eTable 6: Statistics of plasma p-tau217 as stand-alone confirmatory marker of Aβ-positivity**

Clinical accuracy of plasma p-tau217 for different specificity thresholds against Aβ-positivity assessed by either Aβ-PET or CSF, or CSF CSF-only, or Aβ-PET only.

| Outcome | Approach | Accuracy | PPV | NPV | Specificity | Sensitivity |
|---|---|---|---|---|---|---|
| CSF positivity | PET-only | 0.87 [0.86, 0.88] | 0.92 [0.90, 0.94] | 0.85 [0.84, 0.86] | 0.98 [0.97, 0.98] | 0.61 [0.59, 0.64] |
| | Plasma-only | 0.81 [0.80, 0.82] | 0.81 [0.75, 0.87] | 0.81 [0.79, 0.83] | 0.95 [0.92, 0.97] | 0.49 [0.41, 0.57] |
| | Plasma and PET | 0.83 [0.81, 0.85] | 0.99 [0.98, 1.00] | 0.80 [0.78, 0.82] | 1.00 [1.00, 1.00] | 0.44 [0.37, 0.50] |
| Aβ-PET positivity | CSF-only | 0.87 [0.86, 0.88] | 0.62 [0.60, 0.63] | 0.98 [0.97, 0.98] | 0.85 [0.84, 0.86] | 0.93 [0.91, 0.94] |
| | Plasma-only | 0.88 [0.87, 0.89] | 0.76 [0.68, 0.83] | 0.90 [0.89, 0.92] | 0.95 [0.92, 0.97] | 0.60 [0.50, 0.67] |
| | Plasma and CSF | 0.91 [0.89, 0.92] | 0.91 [0.86, 0.95] | 0.90 [0.89, 0.92] | 0.99 [0.97, 0.99] | 0.59 [0.50, 0.66] |

**eTable 7: Statistics for the two-step approach, plasma-only, and invasive test-only strategies**

© 2025 Salvadó G et al. *JAMA Neurology*.

Clinical accuracy of plasma p-tau217 for different approaches including only invasive, only non-invasive or both non-invasive and non-invasive biomarkers with Aβ-PET or CSF as reference.

| | **All (n=964)** | **ADNI (n=240)** | **BioFINDER-2 (n=590)** | **Knight ADRC (n=104)** | **WRAP (n=30)** |
|---|---|---|---|---|---|
| Age, years old | 66.8 (10.5) | 71.5 (6.11) | 64.3 (11.8) | 70.4 (6.66) | 66.1 (6.7) |
| Women, (n%) | 515 (53.4%) | 129 (53.8%) | 310 (52.5%) | 58 (55.8%) | 17 (56.7%) |
| APOE-*ε4* carriers, n(%) | 388 (40.2%) | 73 (30.4%) | 272 (46.1%) | 32 (30.8%) [n=103] | 11 (36.7%) |
| p-tau217 (IA), z-score | 0.567 (1.44) | 0.664 (1.33) | 0.482 (1.38) | 0.689 (1.67) | 0.680 (1.31) |
| p-tau217 (MS), z-score | 0.729 (1.75) | 0.820 (1.48) | 0.693 (1.86) | 0.828 (1.88) | 0.376 (1.14) |
| Aβ-positive, (%n) | 280 (29.0%) | 10 (45.8%) | 156 (26.4%) | 52 (50.0%) | 10 (33.3%) |
| Aβ-PET positive, n(%) | 213 (22.1%) | 107 (44.6%) | 123 (20.8%) | 49 (47.1%) | 6 (20.0%) |
| Centiloids | 11.8 (33.7) | 23.4 (40.2) | 5.70 (30.2) | 19.9 (29.8) | 11.1 (26.4) |
| CSF positive, n(%) | 228 (27.4%) [n=833] | 52 (21.7%) [n=172] | 150 (25.4%) [n=587] | 19 (18.3%) [n=57] | 7 (43.8%) [n=16] |

**eTable 8: Sample characteristics of participants with both immuno-assay and mass-spectrometry based plasma data**

Clinical accuracy of plasma p-tau217 for different approaches including only invasive, only non-invasive or both non-invasive and non-invasive biomarkers with Aβ-PET or CSF as reference. When data is missing, the complete number of participants with that measure is shown between brackets.

| **Plasma platform** | **Accuracy** | **PPV** | **NPV** | **Specificity** | **Sensitivity** |
|---|---|---|---|---|---|
| Immunoassay | 0.82 [0.79,0.84] | 0.80 [0.74,0.86] | 0.82 [0.79,0.85] | 0.95 [0.93,0.97] | 0.49 [0.43,0.55] |
| Mass-spectrometry | 0.88 [0.86,0.90] | 0.85 [0.81,0.90] | 0.88 [0.86,0.91] | 0.95 [0.94,0.97] | 0.69 [0.64,0.75] |

**eTable 9: Statistics for the different plasma assays as stand-alone confirmatory marker of Aβ-positivity**

Clinical accuracy of plasma p-tau217 measured by immunoassay or mass-spectrometry with Aβ-positivity by either Aβ-PET or CSF as reference with a 95% specificity threshold.

© 2025 Salvadó G et al. *JAMA Neurology*.

| Plasma platform | Accuracy | PPV | NPV | Specificity | Sensitivity |
|---|---|---|---|---|---|
| Specificity threshold at 95% | | | | | |
| Immunoassay | 0.84 [0.83, 0.84] | 0.79 [0.75, 0.82] | 0.85 [0.83, 0.87] | 0.95 [0.93, 0.97] | 0.51 [0.41, 0.60] |
| Mass-spectrometry | 0.89 [0.88, 0.89] | 0.84 [0.79, 0.87] | 0.90 [0.88, 0.92] | 0.95 [0.93, 0.97] | 0.70 [0.62, 0.78] |
| Specificity threshold at 97% | | | | | |
| Immunoassay | 0.83 [0.81, 0.84] | 0.83 [0.79, 0.88] | 0.82 [0.81, 0.85] | 0.97 [0.95, 0.98] | 0.41 [0.32, 0.51] |
| Mass-spectrometry | 0.88 [0.85, 0.89] | 0.88 [0.84, 0.91] | 0.88 [0.84, 0.90] | 0.97 [0.95, 0.98] | 0.61 [0.47, 0.68] |
| Specificity threshold at 98% | | | | | |
| Immunoassay | 0.82 [0.81, 0.83] | 0.87 [0.81, 0.91] | 0.81 [0.80, 0.83] | 0.98 [0.97, 0.99] | 0.34 [0.27, 0.43] |
| Mass-spectrometry | 0.86 [0.82, 0.89] | 0.90 [0.87, 0.93] | 0.85 [0.81, 0.89] | 0.98 [0.97, 0.99] | 0.50 [0.33, 0.66] |
| Specificity threshold at 99% | | | | | |
| Immunoassay | 0.80 [0.76, 0.82] | 0.90 [0.86, 0.92] | 0.79 [0.75, 0.81] | 0.99 [0.98, 1.00] | 0.25 [0.07, 0.35] |
| Mass-spectrometry | 0.83 [0.76, 0.87] | 0.93 [0.90, 0.95] | 0.82 [0.76, 0.87] | 0.99 [0.98, 1.00] | 0.35 [0.09, 0.57] |

**eTable 10: Statistics for the different plasma assays as stand-alone confirmatory marker of Aβ-positivity for a subsample with same p-tau217 methods in immunoassay and mass-spectrometry**

Clinical accuracy of plasma p-tau217 measured by immunoassay or mass-spectrometry with Aβ-positivity by either Aβ-PET or CSF as reference with different specificity thresholds. Only BioFINDER-2 individuals were included (n=590).

© 2025 Salvadó G et al. *JAMA Neurology*.

| Threshold | Accuracy | PPV | NPV | Specificity | Sensitivity |
|---|---|---|---|---|---|
| 95% specificity | 0.85 [0.84, 0.85] | 0.80 [0.74, 0.87] | 0.86 [0.84, 0.88] | 0.95 [0.92, 0.97] | 0.56 [0.48, 0.66] |
| 97.5% specificity | 0.83 [0.81, 0.84] | 0.85 [0.79, 0.92] | 0.83 [0.80, 0.85] | 0.97 [0.95, 0.99] | 0.42 [0.32, 0.53] |

**eTable 11: Statistics of plasma p-tau217 as stand-alone confirmatory marker of Aβ-positivity for a subsample with same p-tau217 assay**

Clinical accuracy of plasma p-tau217 with Aβ-positivity by either Aβ-PET or CSF as reference using individuals measured with Lilly's immunoassay at Lund University (n=1,670; ADC, BioFINDER-1, BioFINDER-2, Knight ADRC, PREVENT-AD, SPIN and WRAP).

| Age range | Accuracy | PPV | NPV | Specificity | Sensitivity |
|---|---|---|---|---|---|
| Age <60 (n=448) | 0.87 [0.84, 0.89] | 0.38 [0.27, 0.54] | 0.91 [0.90, 0.92] | 0.95 [0.91, 0.98] | 0.21 [0.08, 0.33] |
| 60≥ Age < 65 (n=337) | 0.79 [0.77, 0.81] | 0.74 [0.59, 0.89] | 0.80 [0.78, 0.82] | 0.95 [0.90, 0.99] | 0.36 [0.24, 0.46] |
| 65≥ Age < 70 (n=438) | 0.83 [0.81, 0.85] | 0.83 [0.72, 0.92] | 0.84 [0.80, 0.86] | 0.95 [0.90, 0.99] | 0.54 [0.40, 0.63] |
| 70≥ Age < 75 (n=383) | 0.78 [0.76, 0.80] | 0.84 [0.75, 0.96] | 0.77 [0.74, 0.81] | 0.95 [0.90, 0.99] | 0.47 [0.36, 0.61] |
| 75≥ Age < 80 (n=276) | 0.74 [0.70, 0.76] | 0.82 [0.70, 0.92] | 0.73 [0.68, 0.77] | 0.95 [0.88, 0.99] | 0.34 [0.16, 0.52] |
| Age ≥80 (n=44) | 0.71 [0.55, 0.86] | 0.93 [0.83, 1.00] | 0.66 [0.51, 0.87] | 0.93 [0.81, 1.00] | 0.51 [0.13, 0.91] |

**eTable 12: Statistics for the different plasma as stand-alone confirmatory marker of Aβ-positivity for different age ranges**

Clinical accuracy of plasma p-tau217 levels for assessing Aβ-positivity by either Aβ-PET or CSF as reference with a 95% specificity threshold. We only included p-tau217 levels, and not age, as predictor in this analysis.

© 2025 Salvadó G et al. *JAMA Neurology*.

**Supplementary references**


1. van der Flier, W. M. *et al.* Optimizing Patient Care and Research: The Amsterdam Dementia Cohort. *Journal of Alzheimer's Disease* **41**, 313–327 (2014).
2. Jagust, W. J. *et al.* The Alzheimer's Disease Neuroimaging Initiative positron emission tomography core. *Alzheimer's and Dementia* **6**, 221–229 (2010).
3. Ellis, K. A. *et al.* The Australian Imaging, Biomarkers and Lifestyle (AIBL) study of aging: methodology and baseline characteristics of 1112 individuals recruited for a longitudinal study of Alzheimer's disease. *Int Psychogeriatr* **21**, 672–687 (2009).
4. Molinuevo, J. L. *et al.* The ALFA project: A research platform to identify early pathophysiological features of Alzheimer's disease. *Alzheimer's and Dementia: Translational Research and Clinical Interventions* **2**, 82–92 (2016).
5. Palmqvist, S. *et al.* Cognitive effects of Lewy body pathology in clinically unimpaired individuals. *Nat Med* **29**, 1971–1978 (2023).
6. Gordon, B. A. *et al.* The relationship between cerebrospinal fluid markers of Alzheimer pathology and positron emission tomography tau imaging. *Brain* **139**, 2249–2260 (2016).
7. Jack, C. R. & Holtzman, D. M. Biomarker Modeling of Alzheimer's Disease. *Neuron* **80**, 1347–1358 (2013).
8. Strikwerda-Brown, C. *et al.* Association of Elevated Amyloid and Tau Positron Emission Tomography Signal With Near-Term Development of Alzheimer Disease Symptoms in Older Adults Without Cognitive Impairment. *JAMA Neurol* **79**, 975 (2022).
9. Alcolea, D. *et al.* The Sant Pau Initiative on Neurodegeneration (SPIN) cohort: A data set for biomarker discovery and validation in neurodegenerative disorders. *Alzheimer's & Dementia: Translational Research & Clinical Interventions* **5**, 597–609 (2019).
10. Pascoal, T. A. *et al.* Discriminative accuracy of the A/T/N scheme to identify cognitive impairment due to Alzheimer's disease. *Alzheimer's & Dementia: Diagnosis, Assessment & Disease Monitoring* **15**, (2023).
11. Johnson, S. C. *et al.* The Wisconsin Registry for Alzheimer's Prevention: A review of findings and current directions. *Alzheimers Dement (Amst)* **10**, 130–142 (2018).
12. Milà-Alomà, M. *et al.* Plasma p-tau231 and p-tau217 as state markers of amyloid-β pathology in preclinical Alzheimer's disease. *Nat Med* **28**, 1797–1801 (2022).
13. Therriault, J. *et al.* Association of Phosphorylated Tau Biomarkers With Amyloid Positron Emission Tomography vs Tau Positron Emission Tomography. *JAMA Neurol* (2022) doi:10.1001/jamaneurol.2022.4485.
14. Groot, C. *et al.* Differential patterns of gray matter volumes and associated gene expression profiles in cognitively-defined Alzheimer's disease subgroups. *Neuroimage Clin* **30**, 102660 (2021).
15. Bourgeat, P. *et al.* Implementing the centiloid transformation for 11C-PiB and β-amyloid 18F-PET tracers using CapAIBL. *Neuroimage* **183**, 387–393 (2018).
16. Salvadó, G. *et al.* Centiloid cut-off values for optimal agreement between PET and CSF core AD biomarkers. *Alzheimers Res Ther* **11**, 1–12 (2019).


© 2025 Salvadó G et al. *JAMA Neurology*.

17. Palmqvist, S. *et al.* Accuracy of brain amyloid detection in clinical practice using cerebrospinal fluid β-Amyloid 42: A cross-validation study against amyloid positron emission tomography. *JAMA Neurol* **71**, 1282–1289 (2014).
18. Therriault, J. *et al.* Determining Amyloid-β Positivity Using [18] F-AZD4694 PET Imaging. *Journal of Nuclear Medicine* **62**, 247–252 (2021).
19. Johnson, S. C. *et al.* Amyloid burden and neural function in people at risk for Alzheimer's Disease. *Neurobiol Aging* **35**, 576–584 (2014).
20. Willemse, E. A. J. *et al.* Comparing CSF amyloid-beta biomarker ratios for two automated immunoassays, Elecsys and Lumipulse, with amyloid PET status. *Alzheimers Dement (Amst)* **13**, e12182 (2021).
21. Milà-Alomà, M. *et al.* Amyloid-β, tau, synaptic, neurodegeneration and glial biomarkers in the preclinical stage of the Alzheimer's continuum. *Alzheimer's and Dementia* 1–14 (2020) doi:10.1002/alz.12131.
22. Hansson, O. *et al.* CSF biomarkers of Alzheimer's disease concord with amyloid-β PET and predict clinical progression: A study of fully automated immunoassays in BioFINDER and ADNI cohorts. *Alzheimer's and Dementia* 1–12 (2018) doi:10.1016/j.jalz.2018.01.010.
23. Gobom, J. *et al.* Validation of the LUMIPULSE automated immunoassay for the measurement of core AD biomarkers in cerebrospinal fluid. *Clinical Chemistry and Laboratory Medicine (CCLM)* **60**, 207–219 (2022).
24. Meyer, P.-F. *et al.* Bi-directional Association of Cerebrospinal Fluid Immune Markers with Stage of Alzheimer's Disease Pathogenesis. *J Alzheimers Dis* **63**, 577–590 (2018).
25. Alcolea, D. *et al.* Agreement of amyloid PET and CSF biomarkers for Alzheimer's disease on Lumipulse. *Ann Clin Transl Neurol* **6**, 1815–1824 (2019).
26. Johnson, S. C. *et al.* Identifying clinically useful biomarkers in neurodegenerative disease through a collaborative approach: the NeuroToolKit. *Alzheimers Res Ther* **15**, 25 (2023).

© 2025 Salvadó G et al. *JAMA Neurology*.
