# Supplementary material for: Plasma Phosphorylated Tau 217 to Identify Preclinical Alzheimer Disease
Source: JAMA Neurol. 2025 Sep 15:e253217. Online ahead of print. doi: 10.1001/jamaneurol.2025.3217 (PMC12558403; doi:10.1001/jamaneurol.2025.3217)
Supplement: Supplement 2. — ADNI, ALFA, and PREVENT-AD Study Groups [file jamaneurol-e253217-s002.pdf]

Supplemental Online Content: Nonauthor Collaborators
*First name, last name, and suffix (if applicable) are required and will appear in PubMed.

*Group Name(s): ADNI, ALFA, and PREVENT-AD Study Groups							
***First Name and Middle Initial(s)**	***Last Name**	***Suffix (eg, Jr, III)**	Academic Degrees	Institution	Location (city, state/province, country)	Role or Contribution, eg, chair, principal investigator	Group (if more than 1 Group listed in the byline) and/or Subgroup (eg, Steering Committee)
Olusegun	Adegoke		MSc	University of Southern California			ADNI
Kedir	Adem Hussen		MS	University of Southern California			ADNI
Paul	Aisen		MD	University of Southern California		ATRI PI	ADNI
Adeyinka	Ajayi		MBBS	MPH Mt. Sinai			ADNI
Hannatu	Amaza			University of Wisconsin			ADNI
Liana G.	Apostolova		MD	Indiana University School of Medicine			ADNI
Miriam	Ashford		PhD	Northern California Institute for Research and Education			ADNI
Omobolanle	Ayo		MBChB	MPH Mt. Sinai			ADNI
Lisa	Barnes		PhD	Rush University			ADNI
Laurel	Beckett		PhD	University of California Davis		Core PI	ADNI
Marie	Bernard		MD	NIA			ADNI
Haley	Bernhardt		BA R. EEG	Washington University St. Louis			ADNI
Virginia	Boatwright		BS	University of Southern California			ADNI
Bret	Borowski		RTR	Mayo Clinic	Rochester		ADNI
Magdalena	Brylska		MS	UPenn School of Medicine			ADNI
Neil	Buckholtz		PhD	National Institute on Aging			ADNI
Yuliana	Cabrera		BS	University of Southern California			ADNI
Nigel J.	Cairns		PhD	MRCPath Washington University St. Louis			ADNI
Maria	Carrillo		PhD	Alzheimer's Association			ADNI
Mark	Choe		BS	Northern California Institute for Research and Education			ADNI
Taylor	Clanton		MPH	University of Southern California			ADNI
Cat	Conti		BA	Northern California Institute for Research and Education			ADNI
Hannah	Craft		MPH	Indiana University School of Medicine			ADNI
Karen	Crawford		MLIS	University of Southern California			ADNI
Sandhitsu	Das		PhD	University of Pennsylvania			ADNI
Charles	DeCarli		MD	University of California Davis			ADNI
Joseph	Di Benedetto		LMSW	Mt. Sinai			ADNI

Supplemental Online Content: Nonauthor Collaborators
*First name, last name, and suffix (if applicable) are required and will appear in PubMed.

***First Name and Middle Initial(s)**	***Last Name**	***Suffix (eg, Jr, III)**	Academic Degrees	Institution	Location (city, state/province, country)	Role or Contribution, eg, chair, principal investigator	Group (if more than 1 Group listed in the byline) and/or Subgroup (eg, Steering Committee)
Adam	Diaz		MS	Northern California Institute for Research and Education			ADNI
Michael	Donohue		PhD	University of Southern California			ADNI
Erin	Drake			Harvard University			ADNI
Claire	Erickson		PhD	University of Pennsylvania			ADNI
Kelley	Faber		MS	CCRC NCRAD/Indiana University School of Medicine			ADNI
Joel	Felmlee		PhD	Mayo Clinic	Rochester		ADNI
Andrea	Fidell		MPH	University of Southern California			ADNI
Derek	Flenniken			Northern California Institute for Research and Education			ADNI
Evan	Fletcher		PhD	University of California Davis			ADNI
Juliet	Fockler			University of California San Francisco			ADNI
Arvin	Forghanian-Arani		PhD	Mayo Clinic	Rochester		ADNI
Tatiana M.	Foroud		PhD	Indiana University School of Medicine		Dir. NCRAD	ADNI
Nick C.	Fox		MD	University College London			ADNI
Richard	Frank		MD PhD	General Electric			ADNI
Erin	Franklin		MS	Washington University St. Louis			ADNI
Matt	Glittenberg			University of Wisconsin			ADNI
Hector	González			University of California San Diego			ADNI
Robert C.	Green		MD PPH	Harvard University (Chair)			ADNI
Joshua	Grill		PhD	University of California Irvine			ADNI
Jeff	Gunter		PhD	Mayo Clinic	Rochester		ADNI
Vanessa	Guzman		PhD	Mt. Sinai			ADNI
Kristin	Harkins			MPH University of Pennsylvania			ADNI
Danielle	Harvey		PhD	University of California Davis		Core PI	ADNI
Caitie	Hedberg			Mayo Clinic	Rochester		ADNI
Lindsey	Hergesheimer		BS	University of Southern California			ADNI
Carole	Ho			Denali Therapeutics			ADNI
Isabella	Hoang			University of Wisconsin			ADNI
John K.	Hsiao		MD	National Institute on Aging			ADNI
Clifford R.	Jack	Jr.	MD	Mayo Clinic	Rochester		ADNI

Supplemental Online Content: Nonauthor Collaborators
*First name, last name, and suffix (if applicable) are required and will appear in PubMed.

*First Name and Middle Initial(s)	*Last Name	*Suffix (eg, Jr, III)	Academic Degrees	Institution	Location (city, state/province, country)	Role or Contribution, eg, chair, principal investigator	Group (if more than 1 Group listed in the byline) and/or Subgroup (eg, Steering Committee)
Jonathan	Jackson		PhD	Massachusetts General Hospital			ADNI
William	Jagust		MD	University of California Berkelely		Core PI	ADNI
Neda	Jahanshad		PhD	University of Southern California School of Medicine			ADNI
Cecily	Jenkins		PhD	University of Southern California			ADNI
Gustavo	Jimenez		MBS	University of Southern California			ADNI
Chengshi	Jin		PhD	University of California San Francisco			ADNI
Taeho	Jo		PhD	Indiana University School of Medicine			ADNI
Zaven	Kachaturian		PhD	Khachaturian Radebaugh & Associates (KRA) Inc			ADNI
Rima	Kaddurah-Daouk		PhD	Duke University/AD Metabolomics Consortium			ADNI
Kejal	Kantarci		MD	Mayo Clinic	Rochester		ADNI
Jason	Karlawish		MD	University of Pennsylvania			ADNI
Zaven	Khachaturian		PhD	Prevent Alzheimer’s Disease 2020		Chair	ADNI
Alexander	Knaack		MS	University of California Davis			ADNI
Robert A.	Koeppe		PhD	University of Michigan			ADNI
Magdalena	Korecka		PhD	UPenn School of Medicine			ADNI
Adrienne	Kormos			Northern California Institute for Research and Education			ADNI
Kaori	Kubo Germano		PhD	Fordham University			ADNI
Winnie	Kwang		MA	University of California San Francisco			ADNI
Kaci	Lacy		MPH	CCRP NCRAD/Indiana University School of Medicine			ADNI
Susan	Landau		PhD	University of California Berkeley		Core PI	ADNI
Emily	Largent		PhD	University of Pennsylvania			ADNI
Edward B.	Lee		MD PhD	University of Pennsylvania		Core PI	ADNI
Virginia M.Y.	Lee		PhD	MBA UPenn School of Medicine			ADNI
Brian	LoPresti			University of Pittsburgh			ADNI
Fabiola	Magana			University of Wisconsin			ADNI
Payam	Mahboubi		MPH	University of Southern California			ADNI
Ian	Malone		PhD	University College London			ADNI
Eliezer	Masliah		MD	NIA			ADNI
Donna	Masterman		MD	Biogen			ADNI

Supplemental Online Content: Nonauthor Collaborators
*First name, last name, and suffix (if applicable) are required and will appear in PubMed.

***First Name and Middle Initial(s)**	***Last Name**	***Suffix (eg, Jr, III)**	Academic Degrees	Institution	Location (city, state/province, country)	Role or Contribution, eg, chair, principal investigator	Group (if more than 1 Group listed in the byline) and/or Subgroup (eg, Steering Committee)
Leonard	Matoush			Mayo Clinic	Rochester		ADNI
Melanie J.	Miller		PhD	Northern California Institute for Research and Education			ADNI
Susan	Molchan		PhD	National Institute on Aging/National Institutes of Health			ADNI
Tom	Montine		MD PhD	University of Washington (Chair)			ADNI
John	Moore-Weiss		PhD	Mayo Clinic	Rochester		ADNI
John	Morris		MD	Washington University St. Louis			ADNI
Scott	Neu		PhD	University of Southern California			ADNI
Kwangsik	Nho		PhD	Indiana University School of Medicine		Core PI	ADNI
Talia M.	Nir		PhD	University of Southern California School of Medicine			ADNI
Rachel	Nosheny		PhD	University of California San Francisco			ADNI
Kelly	Nudelman		PhD	NCRAD/Indiana University School of Medicine			ADNI
Sheila	Ogwang		MPH	University of Southern California			ADNI
Ozioma	Okonkwo		PhD	University of Wisconsin (Core-PI)		Core PI	ADNI
Shaniya	Parkins			Mt Sinai			ADNI
Richard	Perrin		MD PhD	Washington University St. Louis			ADNI
Ronald	Petersen		MD PhD	Mayo Clinic	Rochester	Core PI	ADNI
Jeremy	Pizzola		BA	University of Southern California			ADNI
Zoë	Potter		BA	CCRP NCRAD/Indiana University School of Medicine			ADNI
William	Potter		MD	National Institute of Mental Health			ADNI
Gil	Rabinovici			University of California San Francisco			ADNI
Michael	Rafii		MD PhD	University of Southern California School of Medicine			ADNI
Rema	Raman		PhD	University of Southern California			ADNI
Robert	Reid		PhD	Mayo Clinic	Rochester		ADNI
Calvin	Reyes			Mayo Clinic	Rochester		ADNI
Denise	Reyes			Mayo Clinic	Rochester		ADNI
Shannon L.	Risacher		PhD	Indiana University School of Medicine			ADNI
Monica	Rivera-Mindt		PhD	Fordham University; Mt. Sinai		Core PI	ADNI
Justin	Robison		MS	University of Southern California			ADNI
Stephanie	Rossi Chen		BA	Northern California Institute for Research and Education			ADNI

Supplemental Online Content: Nonauthor Collaborators
*First name, last name, and suffix (if applicable) are required and will appear in PubMed.

***First Name and Middle Initial(s)**	***Last Name**	***Suffix (eg, Jr, III)**	Academic Degrees	Institution	Location (city, state/province, country)	Role or Contribution, eg, chair, principal investigator	Group (if more than 1 Group listed in the byline) and/or Subgroup (eg, Steering Committee)
Laurie	Ryan		PhD	National Institute on Aging			ADNI
Pallavi	Sachdev		PhD	Eisai		Chair 2023-2024	ADNI
Naomi	Saito		MS	University of California Davis			ADNI
Jennifer	Salazar		MBS	University of Southern California			ADNI
Andrew J.	Saykin		PsyD	Indiana University School of Medicine		Core PI	ADNI
Christopher	Schwarz		PhD	Mayo Clinic	Rochester		ADNI
Mai	Seng Thao			University of Wisconsin			ADNI
Matthew	Senjem		MS	Mayo Clinic	Rochester		ADNI
Elizabeth	Shaffer		BS	University of Southern California			ADNI
Leslie M.	Shaw		PhD	University of Pennsylvania			ADNI
Li	Shen		PhD	University of Pennsylvania			ADNI
Nina	Silverberg		PhD	NIA			ADNI
Stephanie	Smith		BS	University of Southern California			ADNI
Peter J.	Snyder		PhD	University of Connecticut			ADNI
Joe	Strong		PhD	University of Wisconsin			ADNI
Sandra	Talavera		MSW	Fordham University			ADNI
Lisa	Taylor-Reinwald		BA HTL	Washington University St. Louis			ADNI
Leon	Thal		MD			Past Investigator	ADNI
Lisa	Thomas			University of Wisconsin			ADNI
Sophia I.	Thomopoulos		BS	University of Southern California School of Medicine			ADNI
Paul	Thompson		PhD	UCLA School of Medicine			ADNI
Arthur W.	Toga		PhD	University of Southern California		Core PI	ADNI
Duygu	Tosun		PhD	University of California San Francisco			ADNI
J.Q.	Trojanowki		MD PhD	UPenn School of Medicine		former Core PI	ADNI
Diana	Truran Sacrey			Northern California Institute for Research and Education			ADNI
Prashanthi	Vemuri		PhD	Mayo Clinic	Rochester		ADNI
Victor	Villemagne			University of Pittsburgh			ADNI
Sarah	Walter		MSc	University of Southern California			ADNI
Yang	Wan		MS	UPenn School of Medicine			ADNI

Supplemental Online Content: Nonauthor Collaborators

*First name, last name, and suffix (if applicable) are required and will appear in PubMed.

*First Name and Middle Initial(s)	*Last Name	*Suffix (eg, Jr, III)	Academic Degrees	Institution	Location (city, state/province, country)	Role or Contribution, eg, chair, principal investigator	Group (if more than 1 Group listed in the byline) and/or Subgroup (eg, Steering Committee)
Chad	Ward			Mayo Clinic	Rochester		ADNI
Caitlin	Webb		BA	University of Southern California			ADNI
Michael	Weiner		MD	University of California San Francisco		PI	ADNI
Trinity	Weisensel			University of Wisconsin			ADNI
Paul A.	Yushkevich		PhD	University of Pennsylvania			ADNI
Caileigh	Zimmerman		MS	University of Southern California			ADNI
Sylvia	Villeneuve		PhD	McGill University	Montreal, QC, CA	Director; Investigator;	PREVENT-AD
Judes	Poirier		PhD	McGill University	Montreal, QC, CA	Co-Director; Investigat	PREVENT-AD
John C.S.	Breitner		MD, MPH	McGill University	Montreal, QC, CA	Director Emeritus; Inve	PREVENT-AD
Mohamed	Badawy		MD	McGill University	Montreal, QC, CA	Investigator; LP/CSF Co	PREVENT-AD
Sylvain	Baillet		PhD	McGill University	Montreal, QC, CA	Investigator	PREVENT-AD
Andrée-Ann	Baril		PhD	McGill University	Montreal, QC, CA	Investigator; Data Anal	PREVENT-AD
**Carolina**	**Minguillon**		**PhD**	**BBRC**	Barcelona, Spain	Collaborator – ALFA stu	ALFA
Ricardo	Aquite Aguilar			BBRC	Barcelona, Spain	Collaborator – ALFA stu	ALFA
Annabella	Beteta Gorriti			BBRC	Barcelona, Spain	Collaborator – ALFA stu	ALFA
Anna	Brugulat-Serrat			BBRC	Barcelona, Spain	Collaborator – ALFA stu	ALFA
Raffaele	Cacciaglia			BBRC	Barcelona, Spain	Collaborator – ALFA stu	ALFA
Lidia	Canals Gispert			BBRC	Barcelona, Spain	Collaborator – ALFA stu	ALFA
Alba	Cañas Martinez			BBRC	Barcelona, Spain	Collaborator – ALFA stu	ALFA
Marta	del Campo Milan			BBRC	Barcelona, Spain	Collaborator – ALFA stu	ALFA
Ruth	Dominguez Iglesias			BBRC	Barcelona, Spain	Collaborator – ALFA stu	ALFA
Sherezade	Fuentes Julian			BBRC	Barcelona, Spain	Collaborator – ALFA stu	ALFA
Patricia	Genius Serra			BBRC	Barcelona, Spain	Collaborator – ALFA stu	ALFA
Armand	González-Escalante			BBRC	Barcelona, Spain	Collaborator – ALFA stu	ALFA
Laura	Hernández Penas			BBRC	Barcelona, Spain	Collaborator – ALFA stu	ALFA
Gema	Huesa Rodriguez			BBRC	Barcelona, Spain	Collaborator – ALFA stu	ALFA
Jordi	Huguet Ninou			BBRC	Barcelona, Spain	Collaborator – ALFA stu	ALFA
Laura	Iglesias Gamaz			BBRC	Barcelona, Spain	Collaborator – ALFA stu	ALFA
Iva	Knezevic			BBRC	Barcelona, Spain	Collaborator – ALFA stu	ALFA
Paula	Marne Alvarez			BBRC	Barcelona, Spain	Collaborator – ALFA stu	ALFA

Supplemental Online Content: Nonauthor Collaborators
*First name, last name, and suffix (if applicable) are required and will appear in PubMed.

***First Name and Middle Initial(s)**	***Last Name**	***Suffix (eg, Jr, III)**	Academic Degrees	Institution	Location (city, state/province, country)	Role or Contribution, eg, chair, principal investigator	Group (if more than 1 Group listed in the byline) and/or Subgroup (eg, Steering Committee)
Tania	Menchón Diaz			BBRC	Barcelona, Spain	Collaborator – ALFA stu	ALFA
Eva	Palacios			BBRC	Barcelona, Spain	Collaborator – ALFA stu	ALFA
Maria	Pascual			BBRC	Barcelona, Spain	Collaborator – ALFA stu	ALFA
Albina	Polo Ballester			BBRC	Barcelona, Spain	Collaborator – ALFA stu	ALFA
Sandra	Pradas Mendez			BBRC	Barcelona, Spain	Collaborator – ALFA stu	ALFA
Blanca	Rodríguez-Fernandez			BBRC	Barcelona, Spain	Collaborator – ALFA stu	ALFA
Laura	Ros Freixedes			BBRC	Barcelona, Spain	Collaborator – ALFA stu	ALFA
Aleix	Sala Vila			BBRC	Barcelona, Spain	Collaborator – ALFA stu	ALFA
Lluis	Solsona Harster			BBRC	Barcelona, Spain	Collaborator – ALFA stu	ALFA
Anna	Soteras Prat			BBRC	Barcelona, Spain	Collaborator – ALFA stu	ALFA
Marc	Vilanova Jaramillo			BBRC	Barcelona, Spain	Collaborator – ALFA stu	ALFA
Natalia	Vilor-Tejedor			BBRC	Barcelona, Spain	Collaborator – ALFA stu	ALFA
