# Supplementary material for: Plasma Phosphorylated Tau 217 to Identify Preclinical Alzheimer Disease
Source: JAMA Neurol. 2025 Sep 15:e253217. Online ahead of print. doi: 10.1001/jamaneurol.2025.3217 (PMC12558403; doi:10.1001/jamaneurol.2025.3217)
Supplement: Supplement 3. — Data sharing statement [file jamaneurol-e253217-s003.pdf]

Data Sharing Statement

Salvadó. Plasma Phosphorylated Tau 217 to Identify Preclinical Alzheimer Disease. *JAMA Neurol*. Published September 15, 2025. doi:10.1001/jamaneurol.2025.3217

Data
Data available: No

Additional Information
Explanation for why data not available: Due to the multicenter design of the study, access to individual participant data from each cohort will have to be made available through the PIs of the respective cohorts. Generally, anonymized data can be shared by request from qualified academic investigators for the purpose of replicating procedures and results presented in the article, if data transfer is in agreement with the data protection regulation at the institution and is approved by the local Ethics Review Board.
